# A 3D non-hydrostatic model for simulating coastal wave transformations: shoaling, diffraction, and refraction

**DOI:** 10.1038/s41598-025-23341-z

**Published:** 2025-11-11

**Authors:** Ali Shirkavand, Kambiz Farrahi-Moghaddam

**Affiliations:** 1 Department of Civil Engineering, VaP.C., Islamic Azad University, Varamin, Iran; 2 Department of Water Engineering, Faculty of Civil and Surveying Engineering, Graduate University of Advanced Technology, Kerman, Iran

**Keywords:** Engineering, Mathematics and computing, Physics

## Abstract

This research develops a 3D non-hydrostatic model to simulate complex free-surface flows, including wave propagation under various conditions. The model discretizes the full 3D Reynolds-averaged Navier–Stokes (RANS) equations using the finite volume method on a staggered computational grid. The grid combines orthogonal cells in the horizontal plane with a curvilinear system conforming to bed and water surface boundaries vertically. The governing equations are solved via a time-splitting pressure-correction approach. Initially, intermediate velocities are computed by addressing advection–diffusion terms, the dynamic pressure gradient, and the water surface gradient in the momentum equations. This is achieved through a time-splitting method with tailored techniques for each component. Subsequently, the provisional velocity fields and pressure correction gradients are incorporated into the continuity equation. A Poisson equation governing pressure correction is then derived. The study introduces a modification to the 3D non-hydrostatic pressure distribution in the surface layer within the pressure correction method, streamlining its implementation compared to similar approaches. With this modification, the model can simulate short-wave propagation in three dimensions using only a few vertical layers. Furthermore, a new approach is implemented for estimating horizontal velocities at vertical velocity locations, reducing computational complexity by increasing the sparsity of the system matrix. The 3D numerical model is validated through simulations of various scenarios. The high level of agreement with experimental and analytical results highlights the model as a reliable and efficient tool for simulating coastal wave processes in practical scenarios.

## Introduction

Computational fluid dynamics (CFD) is widely applied in diverse fields to simulate and optimize fluid-related processes. In energy and food systems, it has been used to evaluate solar dryers for fish products, enhancing drying performance and sustainability^1^. CFD models have improved airflow management and energy efficiency in data centers^2^, and when coupled with machine learning, have optimized combustion systems for lower emissions^3^. In mechanical and biomedical engineering, CFD has contributed to improving exhaust manifold design^4^ and understanding flow behavior in ureteral stents^5^. Applications extend to elevator aerodynamics^6^, sediment-induced clogging in hydraulic filters^7^, eco-friendly spur dike designs^8^, CO₂ separation using ionic liquids^9^, and pollutant dispersion in urban environments^10^. These examples underscore CFD’s versatility across engineering domains. Following the widespread application of CFD across various disciplines, the present study develops a three-dimensional non-hydrostatic model to simulate key coastal wave transformation processes—including shoaling, diffraction, and refraction—over complex bathymetries.

Hydrodynamic flows in nature are intricate and 3D, necessitating the use of appropriate 3D models for accurate simulation of associated phenomena. A critical aspect of these flows is how pressure is taken into account. When simulating time-dependent flows involving a free surface, if the vertical acceleration is significantly smaller than the remaining terms in the pressure equation in the governing equations, it can be disregarded, enabling the assumption of hydrostatic pressure. In such cases, pressure depends on the water surface level, which can be easily determined. However, for specific phenomena, such as those investigated in this research, vertical velocities and accelerations must be carefully considered and accurately calculated in three dimensions.

Various techniques have been developed to simulate non-hydrostatic water surface flows and scenarios involving dynamic boundaries, such as interactions between air and water. In this context, the Volume of Fluid (VOF) approach has been utilized by researchers such as Lin and Liu^11^, Bakhtyar et al.^12^, Liu et al.^13^, Li et al.^14^, and Jin et al.^15^. Christensen and Deigaard^16^ applied the Marker and Cell (MAC) technique, while Wang et al.^17^ and Frantzis et al.^18^ utilised the Level-Set Method in their research. Although these methods can simulate complex water surfaces, they face challenges related to stability and high computational costs, particularly for large-scale applications in coastal areas and rivers^19^. These methods are widely used for two-phase flow simulations and have been validated extensively. However, they are inherently designed to capture detailed air–water interfaces, requiring fine grid resolutions and significant computational resources, even for non-breaking wave cases. While their cost may not be excessively high for simple wave propagation, they are not optimized for scenarios where phase separation is not a primary concern. Addressing the issue of high computational costs is even more critical for 3D simulations.

In addition to fully 3D non-hydrostatic solvers, a variety of alternative modelling approaches have been developed with the aim of reducing computational costs while retaining acceptable accuracy. For example, unstructured finite volume hydrostatic solvers, such as the VOLNA code^20^, are widely used for tsunami generation, propagation, and inundation. Similarly, depth-averaged non-hydrostatic and dispersive models, including modernized Boussinesq-type systems^21^ and modified shallow-water equations for varying seabeds^22^, were proposed to capture key wave transformation processes with the goal of reducing computational demands. However, these simplified approaches, such as shallow-water and Boussinesq-type models, are unable to resolve vertical flow details and fail to accurately simulate the propagation of short waves. By contrast, non-hydrostatic models can reproduce both short-wave propagation and vertical flow structures, even when using only two vertical layers. Notably, Zijlema and Stelling^23^ reported that the computational cost of non-hydrostatic models may even be lower than that of multi-layer Boussinesq-type models, while achieving superior accuracy.

In this research, a 3D model with non-hydrostatic properties was developed to simulate wave shoaling, diffraction, and refraction. The governing equations were solved using the time-splitting approach combined with a pressure-correction projection technique, which consists of two primary steps. The initial phase determined intermediate velocities through the resolution of the advection–diffusion terms, the explicit dynamic pressure gradient, and the gradient of the water surface elevation, utilizing the time splitting approach and suitable methods for each component. This approach significantly reduces computational complexity in multidimensional space, enhancing the model’s efficiency while maintaining accuracy^24–28^. Modifications were made to the governing equations and the computational approach to preserve local momentum balance and ensure the monotonicity of the solution, allowing for the concurrent resolution of the conservation of mass and momentum equations. Considering the critical role of horizontal advection in wave propagation simulations, a Godunov-type shock-capturing scheme was employed to handle these components. To further optimize the discretization process in 3D simulations, a novel method for estimating horizontal velocities at vertical velocity locations was employed. This method reduces the dependency of each computational cell to 15 neighboring cells, compared to the traditional 25-cell framework, effectively reducing the number of non-zero coefficients in the system matrix in the pressure equation system and significantly improving computational performance in 3D applications. In the second step, the intermediate velocity fields, along with the gradient term related to pressure adjustment from the momentum equations, were integrated into the conservation of mass equation, leading to the development and implicit solving of a pressure-adjustment equation, often identified as the Poisson equation. A modification for the distribution of 3D non-hydrostatic pressure in the surface layer was also integrated. This adjustment simplifies the non-hydrostatic pressure distribution in the surface layer compared to other methods and enables the model to capture short wave propagation with just a few vertical layers. Finally, the model was validated through four scenarios: modeling linear standing short waves in a 3D closed basin, simulating sinusoidal wave propagation over a submerged elliptical mound, modeling wave propagation over an elliptical mound on a sloping bed, and simulating long wave resonance in a parabolic basin. This study provides a robust and computationally efficient model for three-dimensional coastal wave dynamics, facilitating enhanced prediction and management of coastal water environments in engineering and environmental applications.

## Governing equations

The Reynolds-averaged Navier–Stokes (RANS) equations in their incompressible and unsteady form were used to simulate the 3D free surface flow. Based on the pressure correction method, if the total pressure *P* in the momentum equations is defined as *P* =  *− ρg(z − η)* + *ρq*, the equations are as follows:

1 $$\frac{\partial u}{{\partial x}} + \frac{\partial v}{{\partial y}} + \frac{\partial w}{{\partial z}} = 0$$

2 $$\frac{\partial u}{{\partial t}} + \frac{\partial uu}{{\partial x}} + \frac{\partial vu}{{\partial y}} + \frac{\partial wu}{{\partial z}} + g\frac{\partial \eta }{{\partial x}} + \frac{\partial q}{{\partial x}} = \frac{\partial }{\partial x}\left( {\nu_{h} \frac{\partial u}{{\partial x}}} \right) + \frac{\partial }{\partial y}\left( {\nu_{h} \frac{\partial u}{{\partial y}}} \right) + \frac{\partial }{\partial z}\left( {\nu_{v} \frac{\partial u}{{\partial z}}} \right)$$

3 $$\frac{\partial v}{{\partial t}} + \frac{\partial uv}{{\partial x}} + \frac{\partial vv}{{\partial y}} + \frac{\partial wv}{{\partial z}} + g\frac{\partial \eta }{{\partial y}} + \frac{\partial q}{{\partial y}} = \frac{\partial }{\partial x}\left( {\nu_{h} \frac{\partial v}{{\partial x}}} \right) + \frac{\partial }{\partial y}\left( {\nu_{h} \frac{\partial v}{{\partial y}}} \right) + \frac{\partial }{\partial z}\left( {\nu_{v} \frac{\partial v}{{\partial z}}} \right)$$

4 $$\frac{\partial w}{{\partial t}} + \frac{\partial uw}{{\partial x}} + \frac{\partial vw}{{\partial y}} + \frac{\partial ww}{{\partial z}} + \frac{\partial q}{{\partial z}} = \frac{\partial }{\partial x}\left( {\nu_{h} \frac{\partial w}{{\partial x}}} \right) + \frac{\partial }{\partial y}\left( {\nu_{h} \frac{\partial w}{{\partial y}}} \right) + \frac{\partial }{\partial z}\left( {\nu_{v} \frac{\partial w}{{\partial z}}} \right)$$

Due to the impermeable nature of the bed, the condition governing vertical velocity at the bed is represented by the following kinematic boundary equation:

5 $$- u\frac{\partial h}{{\partial x}} - v\frac{\partial h}{{\partial y}} = \left. w \right|_{z = - h}$$

The vertical velocity at the water surface was calculated using the kinematic boundary condition (BC) applied there:

6 $$\frac{\partial \eta }{{\partial t}} + u\frac{\partial \eta }{{\partial x}} + v\frac{\partial \eta }{{\partial y}} = \left. w \right|_{z = \eta }$$

The dynamic BC at the water surface is defined by the air pressure. By setting the overall pressure equal to the air pressure at the surface level (*z* = *η*), the dynamic pressure at the surface was calculated:

7 $$P_{a} = - \rho_{0} g(z - \eta ) + \rho_{0} q \Rightarrow q = \frac{{P_{a} }}{{\rho_{0} }} \cong 0$$

In the 3D numerical model, the elevation of the water surface elevation was considered as an unknown variable, resolved simultaneously with other factors. By integrating the conservation of mass equation over the water column and using the Leibniz rule, combined with the application of the kinematic BCs at the bottom layer and the free surface as described in Eqs. (5) and (6), a differential equation for the water surface elevation—or, equivalently, a continuity equation for the water body—was established:

8 $$\frac{\partial \eta }{{\partial t}} + \frac{\partial }{\partial x}\int_{ - h}^{\eta } {udz + \frac{\partial }{\partial y}\int_{ - h}^{\eta } {vdz} } = 0$$

## Treatment of Turbulence

Based on a review of the literature, for the estimation of eddy viscosity in the horizontal direction, the zero-equation Smagorinsky^29^ model is considered appropriate, while for the vertical direction, *k*-*ε* model is employed. Therefore, these models are adopted in this study, with a brief description provided below. More detailed explanations can be found in Rodi^30^.

### Smagorinsky^29^ model

Smagorinsky^29^ approximated the eddy viscosity for horizontal two-dimensional flows as follows:

9 $$\nu_{t} = C_{s}^{2} .\Delta x.\Delta y\sqrt {2\left( {\frac{\partial u}{{\partial x}}} \right)^{2} + 2\left( {\frac{\partial v}{{\partial y}}} \right)^{2} + \left( {\frac{\partial v}{{\partial x}} + \frac{\partial u}{{\partial y}}} \right)^{2} }$$

Where *C*_*s*_ = 0.1–0.2 is the dimensionless Smagorinsky coefficient.

### Standard *k*-*ε* turbulence model

One of the most commonly used two-equation models for turbulence simulation is the *k*-*ε* model. In this study, the *k*-*ε* model is applied to compute the vertical eddy viscosity. The two governing equations of the model are expressed as:

10 $$\frac{\partial k}{{\partial t}} + u_{j} \frac{\partial k}{{\partial x_{j} }} = \frac{\partial }{{\partial x_{j} }}\left[ {\frac{{\nu_{t} }}{{\sigma_{k} }}\frac{\partial k}{{\partial x_{j} }}} \right] + \nu_{t} \frac{{\partial u_{i} }}{{\partial x_{j} }}\left[ {\frac{{\partial u_{i} }}{{\partial x_{j} }} + \frac{{\partial u_{j} }}{{\partial x_{i} }}} \right] - \varepsilon$$

11 $$\frac{\partial \varepsilon }{{\partial t}} + u_{j} \frac{\partial \varepsilon }{{\partial x_{j} }} = \frac{\partial }{{\partial x_{j} }}\left[ {\frac{{\nu_{t} }}{{\sigma_{\varepsilon } }}\frac{\partial \varepsilon }{{\partial x_{j} }}} \right] + c_{1\varepsilon } \frac{\varepsilon }{k}\left[ {\nu_{t} \frac{{\partial u_{i} }}{{\partial x_{j} }}\left[ {\frac{{\partial u_{i} }}{{\partial x_{j} }} + \frac{{\partial u_{j} }}{{\partial x_{i} }}} \right]} \right] - c_{2\varepsilon } \frac{{\varepsilon^{2} }}{k}$$

where *k* is the square root of the turbulent kinetic energy, *ε* is the dissipation rate of turbulent kinetic energy, and ${\nu }_{t}={c}_{\mu }\frac{{k}^{2}}{\varepsilon }$ is the eddy viscosity. The other coefficients of the standard *k*-*ε* model are constant values and are summarized in (Table 1).

**Table 1:** Constant coefficients in the *k*-*ε* turbulence model.

*c*_*1ε*_ (production constant of *ε*)	*c*_*2ε*_ (dissipation constant of *ε*)	*σ*_*k*_ (prandtl number for *k*)	*σ*_*ε*_ (prandtl number for *ε*)	*c*_*µ*_ (eddy viscosity constant)
1.44	1.92	1.00	1.30	0.09

The boundary conditions for the *k*-*ε* model at the free surface and seabed can be applied either as Dirichlet (specified value) or Neumann (specified flux) conditions^31^. For the Dirichlet condition, the wall function approximation is used as follows:

12 $$k = \frac{{u_{*}^{2} }}{{\sqrt {c_{\mu } } }}\,\,\,,\,\,\,\,\,\,\,\,\,\,\varepsilon = \frac{{c_{\mu }^{0.75} k^{1.5} }}{\kappa .z}$$

where *z* is the distance from the wall, *κ* = 0.41 is the von Karman constant, and *u*^*∗*^ is the shear velocity. For the Neumann boundary condition, the flux of turbulent kinetic energy at the surface and bottom is assumed to be zero, while the flux of dissipation rate is calculated as:

13 $$\frac{{\nu_{t} }}{{\sigma_{k} }}\frac{\partial k}{{\partial z}} = 0\,\,\,,\,\,\,\,\,\,\,\,\,\,\frac{{\nu_{t} }}{{\sigma_{\varepsilon } }}\frac{\partial \varepsilon }{{\partial z}} = - c_{\mu }^{0.75} \frac{{\nu_{t} }}{{\sigma_{\varepsilon } }}\frac{{k^{1.5} }}{\kappa .z}$$

For more detailed information, readers are referred to Launder and Spalding^32^ and Burchard^31^.

## Numerical methods

**Fig. 1:**
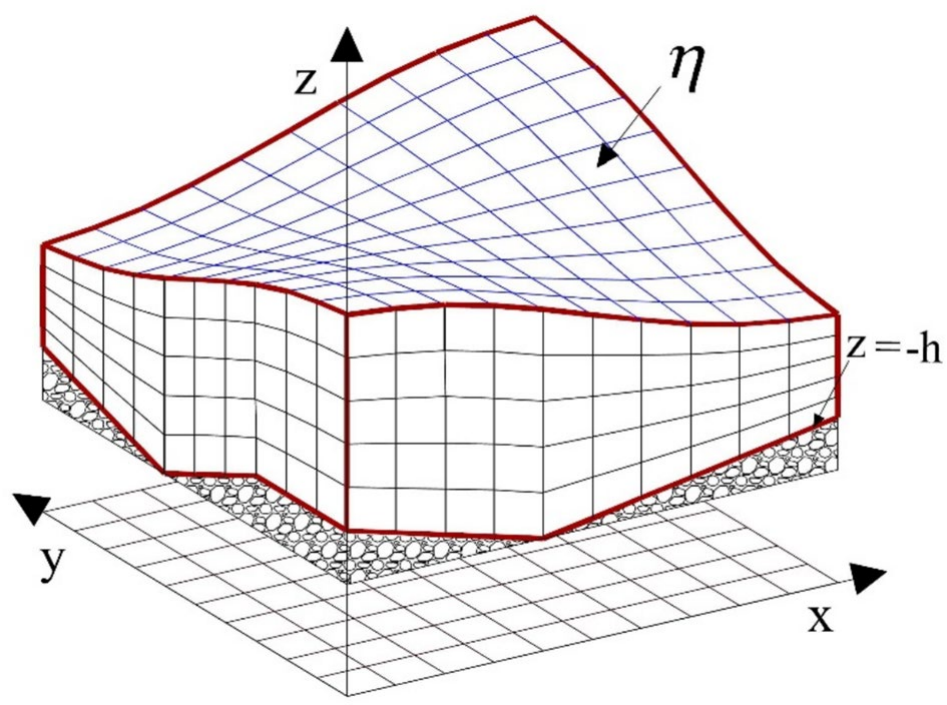
Meshing of the 3D computational domain using a boundary-fitted curvilinear coordinate system.

The governing equations were numerically solved using a computational mesh with Cartesian coordinates in the horizontal plane and curvilinear coordinates aligned with the boundary vertically, as depicted in (Fig. 1).

The horizontal plane of the computational domain was discretized using cells with varying dimensions *Δx* and *Δy*. On the boundary-fitted curvilinear grid at the bed and water surface, the solution domain was divided into several layers, with the layer thicknesses (*d*_*p*_) as follows:

14 $$d_{p} = f_{p} .D = f_{p} .(\eta + h),\,\,\,0 < f_{p} < 1,\,\,\,\sum\limits_{p} {f_{p} } = 1$$

**Fig. 2:**
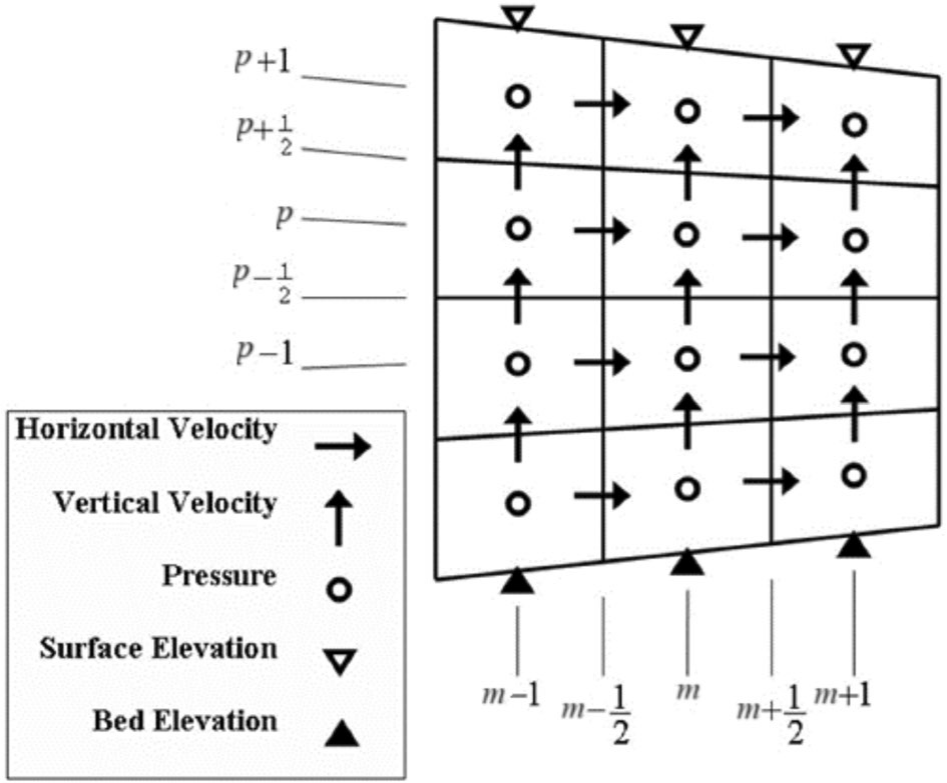
Placement of unknowns in the staggered grid.

The number of vertical cells was kept uniform across the entire grid. In this case, any change in the water surface elevation within a column would alter the vertical dimension of all cells in that column, ensuring that the ratio of the cell height to the total depth remained constant. A standard staggered computational grid was employed for positioning the variables (Fig. 2).

Pressures are assigned to the center of each cell (indices *m,n,p*). Horizontal velocity components *u* are placed at the right and left walls of each cell (indices $m+\frac{1}{2},n,p$ and $m-\frac{1}{2},n,p$), while horizontal velocity components *v* are positioned at the front and back edges (indices $m,n+\frac{1}{2},p$ and $m,n-\frac{1}{2},p$). Vertical velocities are situated at the top and bottom edges (indices $m,n,p+\frac{1}{2}$ and $m,n,p-\frac{1}{2}$). The elevation of the bed (− *h*) and water surface (*η*) are located at the bottom and top of each column and at the center (indices *m,n*). Therefore, the water depth *D* = *η* + *h* is defined at the center of the column, i.e., at the position *m,n*. The water depth at the cell edge was calculated with an upwind method oriented according to the depth-averaged velocity direction^33^. For example, this depth was defined for the location of the horizontal velocity *u* as follows:

15 $$\begin{gathered} \hat{D}_{{m + \tfrac{1}{2},n}} = \left\{ {\begin{array}{*{20}c} {\eta_{m,n} + \min (h_{m,n} ,h_{m + 1,n} )\,\,\,\,\,\,\,\,\,\,\,\,\,\,\,\,\,\,\,\,\,\,\,\,\,\,\,\,\,\,\,\,\,{\text{if}}\,\,\,U_{{m + \tfrac{1}{2},n}} > 0} \\ {\eta_{m + 1,n} + \min (h_{m,n} ,h_{m + 1,n} )\,\,\,\,\,\,\,\,\,\,\,\,\,\,\,\,\,\,\,\,\,\,\,\,\,\,\,\,\,\,{\text{if}}\,\,\,U_{{m + \tfrac{1}{2},n}} < 0} \\ {\max (\eta_{m,n} ,\eta_{m + 1,n} ) + \min (h_{m,n} ,h_{m + 1,n} )\,\,\,\,\,\,\,\,{\text{if}}\,\,\,U_{{m + \tfrac{1}{2},n}} = 0} \\ \end{array} } \right.\,\,\,\,\,\,\,\,\,\,,\,\,\,\,\,\,\,\, \hfill \\ U_{{m + \tfrac{1}{2},n}} = \sum\limits_{p} {f_{p} .u_{{m + \tfrac{1}{2},n,p}} ,\,\,\,} \hat{d}_{{m + \tfrac{1}{2},n,p}} = f_{p} .\hat{D}_{{m + \tfrac{1}{2},n}} \hfill \\ \end{gathered}$$

## Numerical discretization and solving algorithm

In the first step, the horizontal momentum equation *u* was discretized spatially by integrating Eq. (2) over the vertical range from ${z}_{m+\frac{1}{2},n,p-\frac{1}{2}}$ to ${z}_{m+\frac{1}{2},n,p+\frac{1}{2}}$. In the next step, after discretizing Eq. (1) at the location of the velocity *u*, this equation was multiplied by the velocity ${u}_{m+\frac{1}{2},n,p}$ and then subtracted from the equation obtained in the first step. Finally, the result was divided by ${d}_{m+\frac{1}{2},n,p}$, yielding the following relationship:

16 $$\begin{gathered} \frac{{u_{{m + \frac{1}{2},n,p}}^{n + 1} - u_{{m + \frac{1}{2},n,p}}^{n} }}{\Delta t} + \frac{1}{{d_{{m + \tfrac{1}{2},n,p}} }}\left( {\frac{{F_{m + 1,n,p}^{ux} - F_{m,n,p}^{ux} }}{{\Delta x_{{m + \tfrac{1}{2}}} }}} \right) - \frac{{u_{{m + \tfrac{1}{2},n,p}}^{n} }}{{d_{{m + \tfrac{1}{2},n,p}} }}\left( {\frac{{\phi_{m + 1,n,p}^{x} - \phi_{m,n,p}^{x} }}{{\Delta x_{{m + \tfrac{1}{2}}} }}} \right) \hfill \\ \,\,\,\,\,\,\,\, + \frac{1}{{d_{{m + \tfrac{1}{2},n,p}} }}\left( {\frac{{F_{{m + \tfrac{1}{2},n + \tfrac{1}{2},p}}^{uy} - F_{{m + \tfrac{1}{2},n - \tfrac{1}{2},p}}^{uy} }}{{\Delta y_{n} }}} \right) - \frac{{u_{{m + \tfrac{1}{2},n,p}}^{n} }}{{d_{{m + \tfrac{1}{2},n,p}} }}\left( {\frac{{\phi_{{m + \tfrac{1}{2},n + \tfrac{1}{2},p}}^{y} - \phi_{{m + \tfrac{1}{2},n - \tfrac{1}{2},p}}^{y} }}{{\Delta y_{n} }}} \right) \hfill \\ \,\,\,\,\,\,\,\, + \left( {\frac{{u_{{m + \tfrac{1}{2},n,p + \tfrac{1}{2}}}^{adv} .\xi_{{m + \tfrac{1}{2},n,p + \tfrac{1}{2}}} - u_{{m + \tfrac{1}{2},n,p - \tfrac{1}{2}}}^{adv} .\xi_{{m + \tfrac{1}{2},n,p - \tfrac{1}{2}}} }}{{d_{{m + \tfrac{1}{2},n,p}} }}} \right) - u_{{m + \tfrac{1}{2},n,p}}^{n} \left( {\frac{{\xi_{{m + \tfrac{1}{2},n,p + \tfrac{1}{2}}} - \xi_{{m + \tfrac{1}{2},n,p - \tfrac{1}{2}}} }}{{d_{{m + \tfrac{1}{2},n,p}} }}} \right) \hfill \\ \,\,\,\,\,\,\,\, + g\frac{{\eta_{m + 1,n} - \eta_{m,n} }}{{\Delta x_{{m + \tfrac{1}{2}}} }} + \frac{1}{{d_{{m + \tfrac{1}{2},n,p}} }}\left( {\frac{{d_{m + 1,n,p} .q_{m + 1,n,p}^{n + \theta } - d_{m,n,p} .q_{m,n,p}^{n + \theta } }}{{\Delta x_{{m + \tfrac{1}{2}}} }}} \right) \hfill \\ \,\,\,\,\,\,\,\,\,\, - \frac{{q_{{m + \frac{1}{2},n,p + \frac{1}{2}}}^{n + \theta } }}{{d_{{m + \tfrac{1}{2},n,p}} }}\,.\,\frac{{z_{{m + 1,n,p + \frac{1}{2}}} - z_{{m,n,p + \frac{1}{2}}} }}{{\Delta x_{{m + \tfrac{1}{2}}} }}\, + \frac{{q_{{m + \frac{1}{2},n,p - \frac{1}{2}}}^{n + \theta } }}{{d_{{m + \tfrac{1}{2},n,p}} }}\,.\,\,\frac{{z_{{m + 1,n,p - \frac{1}{2}}} - z_{{m,n,p - \frac{1}{2}}} }}{{\Delta x_{{m + \tfrac{1}{2}}} }} \hfill \\ \,\,\,\,\,\,\,\,\,\,\,\,\, = ... \hfill \\ \end{gathered}$$

where:

17 $$\phi_{m,n,p}^{x} = \tfrac{1}{2}\left( {\phi_{{m + \tfrac{1}{2},n,p}}^{x} + \phi_{{m - \tfrac{1}{2},n,p}}^{x} } \right)\,\,\,\,\,\,,\,\,\,\,\,\,\,\,\,\,\,\,\,\phi_{{m + \tfrac{1}{2},n,p}}^{x} = \hat{d}_{{m + \tfrac{1}{2},n,p}} .u_{{m + \tfrac{1}{2},n,p}}$$

18 $$\phi_{{m + \tfrac{1}{2},n + \tfrac{1}{2},p}}^{y} = \tfrac{1}{2}\left( {\phi_{{m + 1,n + \tfrac{1}{2},p}}^{y} + \phi_{{m,n + \tfrac{1}{2},p}}^{y} } \right)\,\,\,\,\,\,,\,\,\,\,\,\,\,\,\,\,\,\,\,\phi_{{m + 1,n + \tfrac{1}{2},p}}^{y} = \hat{d}_{{m + 1,n + \tfrac{1}{2},p}} .v_{{m + 1,n + \tfrac{1}{2},p}} \,\,$$

19 $$\xi_{{m + \tfrac{1}{2},n,p + \tfrac{1}{2}}} = \tfrac{1}{2}(\xi_{{m + 1,n,p + \tfrac{1}{2}}} + \xi_{{m,n,p + \tfrac{1}{2}}} )\,\,,\,\,\,\xi_{{m,n,p + \tfrac{1}{2}}} = w_{{m,n,p + \tfrac{1}{2}}} - \frac{{\partial z_{{m,n,p + \tfrac{1}{2}}} }}{\partial t} - u_{{m,n,p + \tfrac{1}{2}}} \frac{{\partial z_{{m,n,p + \tfrac{1}{2}}} }}{\partial x}$$

20 $$q^{n + \theta } = \theta .q^{n + 1} + (1 - \theta ).q^{n} = q^{n} + \theta .(q^{n + 1} - q^{n} ) = q^{n} + \theta .\Delta q$$

21 $$u_{{m + \tfrac{1}{2},n,p + \tfrac{1}{2}}}^{adv} = \left\{ {\begin{array}{*{20}c} {u_{{m + \tfrac{1}{2},n,p}} \,\,\,\,\,\,\,\,\,\,\,\,\,\,{\text{if}}\,\,\,\,\,\,\,\,\xi_{{m + \tfrac{1}{2},n,p + \tfrac{1}{2}}} > 0} \\ {u_{{m + \tfrac{1}{2},n,p + 1}} \,\,\,\,\,\,\,\,\,\,{\text{if}}\,\,\,\,\,\,\,\,\xi_{{m + \tfrac{1}{2},n,p + \tfrac{1}{2}}} < 0} \\ \end{array} } \right.$$

The calculation method for ${u}_{m,n, p+\frac{1}{2}}$ (in Eq. 19) and similar parameters, which involves determining horizontal velocities at vertical velocity locations, will be described in the next section. In the present study, *θ* = 1 was considered. The discretization of Eq. (3) was performed similarly to the horizontal momentum equation *u*. By integrating Eqs. (1) and (3) over the vertical range from ${z}_{m,n+\frac{1}{2},p-\frac{1}{2}}$ to ${z}_{m,n+\frac{1}{2},p+\frac{1}{2}}$ and at the location of the horizontal velocities *v*, the final result of the discretization is as follows:

22 $$\begin{gathered} \frac{{v_{{m,n + \tfrac{1}{2},p}}^{n + 1} - v_{{m,n + \tfrac{1}{2},p}}^{n} }}{\Delta t} + \frac{1}{{d_{{m,n + \tfrac{1}{2},p}} }}\left( {\frac{{F_{{m + \tfrac{1}{2},n + \tfrac{1}{2},p}}^{vx} - F_{{m - \tfrac{1}{2},n + \tfrac{1}{2},p}}^{vx} }}{{\Delta x_{m} }}} \right) \hfill \\ \,\,\,\,\,\,\,\,\,\, - \frac{{v_{{m,n + \tfrac{1}{2},p}}^{n} }}{{d_{{m,n + \tfrac{1}{2},p}} }}\left( {\frac{{\phi_{{m + \tfrac{1}{2},n + \tfrac{1}{2},p}}^{x} - \phi_{{m - \tfrac{1}{2},n + \tfrac{1}{2},p}}^{x} }}{{\Delta x_{m} }}} \right) + \frac{1}{{d_{{m,n + \tfrac{1}{2},p}} }}\left( {\frac{{F_{m,n + 1,p}^{vy} - F_{m,n,p}^{vy} }}{{\Delta y_{{n + \tfrac{1}{2}}} }}} \right) \hfill \\ \,\,\,\,\,\,\,\,\,\, - \frac{{v_{{m,n + \tfrac{1}{2},p}}^{n} }}{{d_{{m,n + \tfrac{1}{2},p}} }}\left( {\frac{{\phi_{m,n + 1,p}^{y} - \phi_{m,n,p}^{y} }}{{\Delta y_{{n + \tfrac{1}{2}}} }}} \right) + \left( {\frac{{v_{{m,n + \tfrac{1}{2},p + \tfrac{1}{2}}}^{adv} .\xi_{{m,n + \tfrac{1}{2},p + \tfrac{1}{2}}} - v_{{m,n + \tfrac{1}{2},p - \tfrac{1}{2}}}^{adv} .\xi_{{m,n + \tfrac{1}{2},p - \tfrac{1}{2}}} }}{{d_{{m,n + \tfrac{1}{2},p}} }}} \right) \hfill \\ \,\,\,\,\,\,\,\,\,\, - v_{{m,n + \tfrac{1}{2},p}}^{n} \left( {\frac{{\xi_{{m,n + \tfrac{1}{2},p + \tfrac{1}{2}}} - \xi_{{m,n + \tfrac{1}{2},p - \tfrac{1}{2}}} }}{{d_{{m,n + \tfrac{1}{2},p}} }}} \right)\, + g\frac{{\eta_{m,n + 1} - \eta_{m,n} }}{{\Delta y_{{n + \tfrac{1}{2}}} }} \hfill \\ \,\,\,\,\,\,\,\,\,\, + \frac{1}{{d_{{m,n + \tfrac{1}{2},p}} }}\left( {\frac{{d_{m,n + 1,p} .q_{m,n + 1,p}^{n + \theta } - d_{m,n,p} .q_{m,n,p}^{n + \theta } }}{{\Delta y_{{n + \tfrac{1}{2}}} }}} \right) \hfill \\ \,\,\,\,\,\,\,\,\,\, - \frac{{q_{{m,n + \frac{1}{2},p + \frac{1}{2}}}^{n + \theta } }}{{d_{{m,n + \tfrac{1}{2},p}} }}\,.\,\frac{{z_{{m,n + 1,p + \frac{1}{2}}} - z_{{m,n,p + \frac{1}{2}}} }}{{\Delta y_{{n + \tfrac{1}{2}}} }}\, + \frac{{q_{{m,n + \frac{1}{2},p - \frac{1}{2}}}^{n + \theta } }}{{d_{{m,n + \tfrac{1}{2},p}} }}\,.\,\,\frac{{z_{{m,n + 1,p - \frac{1}{2}}} - z_{{m,n,p - \frac{1}{2}}} }}{{\Delta y_{{n + \tfrac{1}{2}}} }} = ... \hfill \\ \end{gathered}$$

where:

23 $$v_{{m,n + \tfrac{1}{2},p + \tfrac{1}{2}}}^{adv} = \left\{ {\begin{array}{*{20}c} {v_{{m,n + \tfrac{1}{2},p}} \,\,\,\,\,\,\,\,\,\,\,{\text{if}}\,\,\,\,\,\,\,\,\omega_{{m,n + \tfrac{1}{2},p + \tfrac{1}{2}}} > 0} \\ {v_{{m,n + \tfrac{1}{2},p + 1}} \,\,\,\,\,\,\,\,\,{\text{if}}\,\,\,\,\,\,\,\,\omega_{{m,n + \tfrac{1}{2},p + \tfrac{1}{2}}} < 0} \\ \end{array} } \right.$$

Similarly, the same approach was used for the discretization of Eq. (4). Therefore, by integrating Eqs. (1) and (4) over the vertical range from *z*_*m,n,p*_ to *z*_*m,n,p*+*1*_ and combining them as previously described, the following relation was obtained:

24 $$\begin{gathered} \frac{{w_{{m,n,p + \tfrac{1}{2}}}^{n + 1} - w_{{m,n,p + \tfrac{1}{2}}}^{n} }}{\Delta t} + \frac{1}{{d_{{m,n,p + \tfrac{1}{2}}} }}\left( {\frac{{w_{{m + \tfrac{1}{2},n,p + \tfrac{1}{2}}}^{adv} .\phi_{{m + \tfrac{1}{2},n,p + \tfrac{1}{2}}}^{x} - w_{{m - \tfrac{1}{2},n,p + \tfrac{1}{2}}}^{adv} .\phi_{{m - \tfrac{1}{2},n,p + \tfrac{1}{2}}}^{x} }}{{\Delta x_{m} }}} \right) \hfill \\ \,\,\,\, - \frac{{w_{{m,n,p + \tfrac{1}{2}}}^{n} }}{{d_{{m,n,p + \tfrac{1}{2}}} }}\left( {\frac{{\phi_{{m + \tfrac{1}{2},n,p + \tfrac{1}{2}}}^{x} - \phi_{{m - \tfrac{1}{2},n,p + \tfrac{1}{2}}}^{x} }}{{\Delta x_{m} }}} \right) + \frac{1}{{d_{{m,n,p + \tfrac{1}{2}}} }}\left( {\frac{{w_{{m,n + \tfrac{1}{2},p + \tfrac{1}{2}}}^{adv} .\phi_{{m,n + \tfrac{1}{2},p + \tfrac{1}{2}}}^{y} - w_{{m,n - \tfrac{1}{2},p + \tfrac{1}{2}}}^{adv} .\phi_{{m,n - \tfrac{1}{2},p + \tfrac{1}{2}}}^{y} }}{{\Delta y_{n} }}} \right) \hfill \\ \,\,\,\, - \frac{{w_{{m,n,p + \tfrac{1}{2}}}^{n} }}{{d_{{m,n,p + \tfrac{1}{2}}} }}\left( {\frac{{\phi_{{m,n + \tfrac{1}{2},p + \tfrac{1}{2}}}^{y} - \phi_{{m,n - \tfrac{1}{2},p + \tfrac{1}{2}}}^{y} }}{{\Delta y_{n} }}} \right)\, + \left( {\frac{{w_{m,n,p + 1}^{adv} .\xi_{m,n,p + 1} - w_{m,n,p}^{adv} .\xi_{m,n,p} }}{{d_{{m,n,p + \tfrac{1}{2}}} }}} \right) \hfill \\ \,\,\,\, - w_{{m,n,p + \tfrac{1}{2}}}^{n} \left( {\frac{{\xi_{m,n,p + 1} - \xi_{m,n,p} }}{{d_{{m,n,p + \tfrac{1}{2}}} }}} \right) + \frac{{q_{m,n,p + 1}^{n + \theta } - q_{m,n,p}^{n + \theta } }}{{d_{{m,n,p + \tfrac{1}{2}}} }} \hfill \\ \,\,\,\,\,\,\,\,\,\,\,\,\, = ... \hfill \\ \end{gathered}$$

where:

25 $$w_{{m + \tfrac{1}{2},n,p + \tfrac{1}{2}}}^{adv} = \left\{ {\begin{array}{*{20}c} {w_{{m,n,p + \tfrac{1}{2}}} \,\,\,\,\,\,\,\,\,\,\,{\text{if}}\,\,\,\,\,\,\,\,\phi_{{m + \tfrac{1}{2},n,p + \tfrac{1}{2}}}^{x} > 0} \\ {w_{{m + 1,n,p + \tfrac{1}{2}}} \,\,\,\,\,\,\,\,\,{\text{if}}\,\,\,\,\,\,\,\,\phi_{{m + \tfrac{1}{2},n,p + \tfrac{1}{2}}}^{x} < 0} \\ \end{array} } \right.$$

26 $$w_{{m,n + \tfrac{1}{2},p + \tfrac{1}{2}}}^{adv} = \left\{ {\begin{array}{*{20}c} {w_{{m,n,p + \tfrac{1}{2}}} \,\,\,\,\,\,\,\,\,\,\,{\text{if}}\,\,\,\,\,\,\,\,\phi_{{m,n + \tfrac{1}{2},p + \tfrac{1}{2}}}^{y} > 0} \\ {w_{{m,n + 1,p + \tfrac{1}{2}}} \,\,\,\,\,\,\,\,\,{\text{if}}\,\,\,\,\,\,\,\,\phi_{{m,n + \tfrac{1}{2},p + \tfrac{1}{2}}}^{y} < 0} \\ \end{array} } \right.$$

27 $$w_{m,n,p + 1}^{adv} = \left\{ {\begin{array}{*{20}c} {w_{{m,n,p + \tfrac{1}{2}}} \,\,\,\,\,\,\,\,\,\,\,{\text{if}}\,\,\,\,\,\,\,\,\omega_{m,n,p + 1} > 0} \\ {w_{{m,n,p + \tfrac{3}{2}}} \,\,\,\,\,\,\,\,\,{\text{if}}\,\,\,\,\,\,\,\,\omega_{m,n,p + 1} < 0} \\ \end{array} } \right.$$

Equation (6) was discretized as follows:

28 $$\begin{gathered} \frac{{\eta_{m,n}^{n + 1} - \eta_{m,n}^{n} }}{\Delta t} + \frac{{\hat{D}_{{m + \tfrac{1}{2},n}}^{n} .U_{{m + \tfrac{1}{2},n}}^{n + \theta } - \hat{D}_{{m - \tfrac{1}{2},n}}^{n} .U_{{m - \tfrac{1}{2},n}}^{n + \theta } }}{{\Delta x_{m} }} + \frac{{\hat{D}_{{m,n + \tfrac{1}{2}}}^{n} .V_{{m,n + \tfrac{1}{2}}}^{n + \theta } - \hat{D}_{{m,n - \tfrac{1}{2}}}^{n} .V_{{m,n - \tfrac{1}{2}}}^{n + \theta } }}{{\Delta y_{n} }} = 0\, \hfill \\ V_{{m + \tfrac{1}{2},n}} = \sum\limits_{p} {f_{p} .v_{{m,n + \tfrac{1}{2},p}} \,} \hfill \\ \end{gathered}$$

This research utilized a pressure correction projection technique to solve the equations. In the first step of this method, the intermediate horizontal and vertical velocities were computed using the three momentum equations. At this stage, the pressure terms were computed using the values from the preceding time step:

29 $$\begin{gathered} \frac{{u_{{m + \tfrac{1}{2},n,p}}^{{\text{int}}} - u_{{m + \tfrac{1}{2},n,p}}^{n} }}{\Delta t} + ... + \frac{{\partial q^{n} }}{\partial x} = ... \hfill \\ \frac{{v_{{m,n + \tfrac{1}{2},p}}^{{\text{int}}} - v_{{m,n + \tfrac{1}{2},p}}^{n} }}{\Delta t} + ... + \frac{{\partial q^{n} }}{\partial y} = ... \hfill \\ \frac{{w_{{m,n,p + \tfrac{1}{2}}}^{{\text{int}}} - w_{{m,n,p + \tfrac{1}{2}}}^{n} }}{\Delta t} + ... + \frac{{\partial q^{n} }}{\partial z} = ... \hfill \\ \end{gathered}$$

Then, in the next step, the remaining pressure gradient terms were used to update the intermediate velocities:

30 $$\begin{gathered} \frac{{u_{{m + \tfrac{1}{2},n,p}}^{n + 1} - u_{{m + \tfrac{1}{2},n,p}}^{{\text{int}}} }}{\Delta t} + \theta \frac{\partial (\Delta q)}{{\partial x}} = 0 \hfill \\ \frac{{v_{{m,n + \tfrac{1}{2},p}}^{n + 1} - v_{{m,n + \tfrac{1}{2},p}}^{{\text{int}}} }}{\Delta t} + \theta \frac{\partial (\Delta q)}{{\partial y}} = 0 \hfill \\ \frac{{w_{{m,n,p + \tfrac{1}{2}}}^{n + 1} - w_{{m,n,p + \tfrac{1}{2}}}^{{\text{int}}} }}{\Delta t} + \theta \frac{\partial (\Delta q)}{{\partial z}} = 0 \hfill \\ \end{gathered}$$

In the subsequent phase of the pressure-correction projection approach, the intermediate velocities obtained from Eq. (30) along with the gradient term of the pressure correction were substituted into the conservation of mass equation. This yielded a Poisson equation for pressure-correction, which was then solved.

**Fig. 3:**
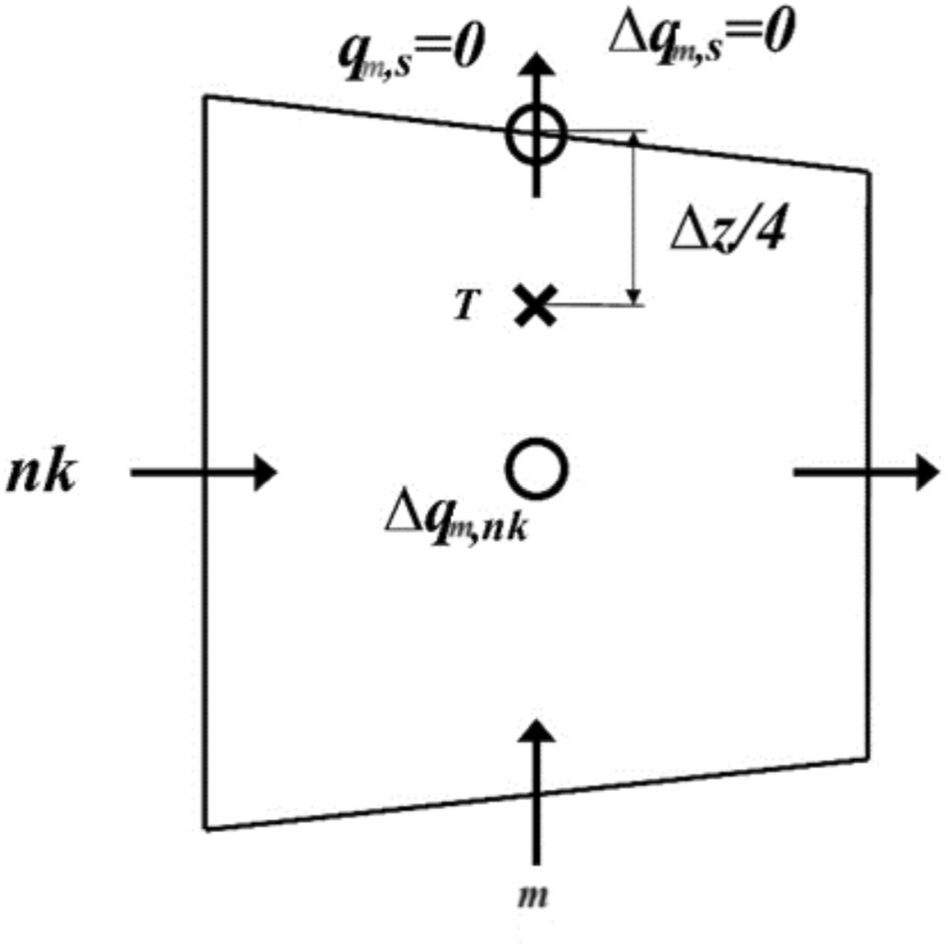
Configuration of parameters in the surface layer.

Referring to Eq. (7), the dynamic pressure on the free surface is set to zero (see Fig. 3). The vertical momentum equation for the top portion of the surface layer is formulated as follows:

31 $$\frac{{w_{m,n,T}^{n + 1} - w_{m,n,T}^{{\text{int}}} }}{\Delta t} + \theta \left( {\frac{{\Delta q_{m,n,s} - \Delta q_{m,n,np} }}{{\Delta z_{m,n,np} /2}}} \right) = 0\,\,\, \Rightarrow \,\,\frac{{w_{m,n,T}^{n + 1} - w_{m,n,T}^{{\text{int}}} }}{\Delta t} + 2.\theta \left( {\frac{{\Delta q_{m,n,np} }}{{\Delta z_{m,n,np} }}} \right) = 0$$

*W*_*m,n,T*_ was estimated using a third-degree polynomial interpolation:

32 $$w_{m,n,T} = b_{1} .w_{{m,n,np + \frac{1}{2}}} + b_{2} .w_{{m,n,np - \frac{1}{2}}} + b_{3} .w_{{m,n,np - \frac{3}{2}}}$$

The coefficients above were calculated based on the weighted layer thickness. Finally, after solving the pressure-correction equation, the obtained *Δq* values were substituted into Eq. (30) to calculate the final velocities *u*, *v*, and *w*, and *η* was calculated from Eq. (28).

## Treatment of bottom friction

In the simulation of wave propagation in the coastal region, bottom roughness becomes important and must be incorporated into the horizontal momentum equation as the bed shear stress, *τ*_*b*_. This bed shear stress term is applied implicitly and only to the bottom layer velocity, as follows:

33 $$\frac{{u_{{m + \tfrac{1}{2},1,p}}^{n + 1} - u_{{m + \tfrac{1}{2},1,p}}^{n} }}{\Delta t} + C_{f} \frac{{(U_{{m + \tfrac{1}{2},1}}^{n} )^{2} }}{{d_{{m + \tfrac{1}{2},1,p}} .\left| {u_{{m + \tfrac{1}{2},1,p}}^{n} } \right|}}u_{{m + \tfrac{1}{2},1,p}}^{n + 1} = 0\,\,\,\,\,,\,\,\,\,\,\,\,\,C_{f} = \frac{{g.n^{2} }}{{D_{{m + \tfrac{1}{2},1}}^{{\tfrac{1}{3}}} }}$$

where *C*_*f*_ is the dimensionless bottom friction coefficient and *n* is the Manning roughness coefficient.

## Determining horizontal velocities at vertical velocity points

As shown in equations like Eq. (19), the use of a boundary-fitted grid and the finite volume method results in terms involving horizontal velocities located at positions where vertical velocities are defined (e.g., ${u}_{m,n, p+\frac{1}{2}}$), but horizontal velocities are not. To address this issue in 3D simulations, this study proposes a novel approach. In this approach, the horizontal velocity values, such as ${u}_{m,n, p+\frac{1}{2}}$, are estimated using the following relation:

34 $$\frac{{u_{{m,n,p + \tfrac{1}{2}}}^{n + 1} - u_{{m,n,p + \tfrac{1}{2}}}^{*} }}{\Delta t} + \theta \frac{1}{{d_{{m,n,p + \tfrac{1}{2}}} }}\int_{{z_{m,n,p} }}^{{z_{m,n,p + 1} }} {\left( {\frac{\partial (\Delta q)}{{\partial x}}} \right)_{{m,n,p + \tfrac{1}{2}}} dz} = 0$$

${u}_{m,n,p+\frac{1}{2}}^{*}$, calculated as the average of the four surrounding intermediate horizontal velocities, as shown below:

35 $$u_{{m,n,p + \tfrac{1}{2}}}^{*} = e_{{m,n,p + \tfrac{1}{2}}}^{1} (u_{{m - \tfrac{1}{2},n,p}}^{*} + u_{{m + \tfrac{1}{2},n,p}}^{*} ) + e_{{m,n,p + \tfrac{1}{2}}}^{2} (u_{{m - \tfrac{1}{2},n,p + 1}}^{*} + u_{{m + \tfrac{1}{2},n,p + 1}}^{*} )$$

*e* coefficients, for a grid with unequal layer ratios, are calculated as follows:

36 $$e_{{m,p + \tfrac{1}{2}}}^{1} = \frac{1}{2}\frac{{f_{p + 1} }}{{f_{p} + f_{p + 1} }}\,\,\,\,\,\,\,\,\,\,\,,\,\,\,\,\,\,\,\,\,\,\,\,\,\,\,e_{{m,p + \tfrac{1}{2}}}^{2} = \frac{1}{2}\frac{{f_{p} }}{{f_{p} + f_{p + 1} }}$$

The advantage of this method lies in applying averaging only to the explicit terms, while directly incorporating the dynamic pressure correction gradient into the implicit terms of the equations. This approach reduces the dependency of each cell on only 15 surrounding cells, significantly decreasing the computational effort required to solve the system of linear equations. In contrast, extending classical methods^34,35^ to the three-dimensional case results in each cell depending on 25 surrounding cells.

## Computational techniques for handling advection terms

When modeling the behavior of highly nonlinear waves, such as during wave breaking, the term representing the advection (convection) of momentum is critical. The method used to solve it greatly influences the accuracy and reliability of the outcomes. Among the various convective terms in the momentum equations, the convection of horizontal velocities along the x-axis and y-axis is the most significant. As a result, the method for solving these terms varies from the methods used for the other advection terms. There are various methods for solving the advection term, each differing in temporal and spatial accuracy. In this study, to achieve second-order temporal accuracy, a predictor–corrector approach was employed. During the prediction step, the fluxes in the convection equation were computed with the Godunov scheme by accurately solving the Riemann initial value problem. In the correction step, a backward approach was utilized. This method, applied by Yekta and Banihashemi^36^ to shallow water equations, was adapted here for a 3D non-hydrostatic model.

Given the application of the time-splitting algorithm in this study, the horizontal advection term in the momentum equation was split as follows:

37 $$\frac{\partial u}{{\partial t}} + \frac{1}{d}\frac{\partial F}{{\partial x}} = 0\,\,\,,\,\,\,F = u^{2} d$$

In the prediction step, the above equation was discretized:

38 $$\frac{{u_{{m + \frac{1}{2},n,p}}^{p} - u_{{m + \frac{1}{2},n,p}}^{n} }}{\Delta t} + \frac{1}{{d_{{m + \frac{1}{2},n,p}} }}\frac{{F_{m + 1,n,p}^{p} - F_{m,n,p}^{p} }}{\Delta x} = 0$$

The Riemann problem for Eq. (38) is defined by the following relation:

39 $$u^{n} (x,t) = \left\{ {\begin{array}{*{20}c} {u_{Le} = u_{{m - \tfrac{1}{2},n,p}}^{n} \,\,\,\,\,\,\,\,\,\,\,\,{\text{if}}\,\,\,\,\,\,\,\,x \le x_{m} } \\ {u_{Ri} = u_{{m + \tfrac{1}{2},n,p}}^{n} \,\,\,\,\,\,\,\,\,\,\,\,{\text{if}}\,\,\,\,\,\,\,\,x > x_{m} } \\ \end{array} } \right.$$

In the prediction step, the fluxes were evaluated with the Godunov method by solving the Riemann problem as follows:

40 $$F_{m,n,p}^{p} = \left\{ {\begin{array}{*{20}l} {\hat{d}_{{m - \tfrac{1}{2},n,p}} u_{Le}^{2} \,\,\,\,\,\,\,\,\,\,\,\,\,\,\,\,\,\,\,\,\,\,\,\,\,\,\,\,\,\,\,\,\,\,\,\,\,\,{\text{if}}\,\,\,\,\,\,\,\,\,\,\,u_{Le} > u_{Ri} \,,\,u_{Le} + u_{Ri} > 0} \hfill \\ {\hat{d}_{{m - \tfrac{1}{2},n,p}} u_{Le}^{2} = \hat{d}_{{m + \tfrac{1}{2},n,p}} u_{Ri}^{2} \,\,\,\,\,\,\,\,\,\,{\text{if}}\,\,\,\,\,\,\,\,\,\,\,u_{Le} > u_{Ri} \,,\,u_{Le} + u_{Ri} = 0} \hfill \\ {\hat{d}_{{m + \tfrac{1}{2},n,p}} u_{Ri}^{2} \,\,\,\,\,\,\,\,\,\,\,\,\,\,\,\,\,\,\,\,\,\,\,\,\,\,\,\,\,\,\,\,\,\,\,\,\,\,{\text{if}}\,\,\,\,\,\,\,\,\,\,\,u_{Le} > u_{Ri} \,,\,u_{Le} + u_{Ri} < 0} \hfill \\ {\hat{d}_{{m - \tfrac{1}{2},n,p}} u_{Le}^{2} = \hat{d}_{{m + \tfrac{1}{2},n,p}} u_{Ri}^{2} \,\,\,\,\,\,\,\,\,\,{\text{if}}\,\,\,\,\,\,\,\,\,\,\,u_{Le} = u_{Ri} } \hfill \\ {\hat{d}_{{m - \tfrac{1}{2},n,p}} u_{Le}^{2} \,\,\,\,\,\,\,\,\,\,\,\,\,\,\,\,\,\,\,\,\,\,\,\,\,\,\,\,\,\,\,\,\,\,\,\,\,{\text{if}}\,\,\,\,\,\,\,\,\,\,\,u_{Le} < u_{Ri} \,,\,u_{Le} > 0} \hfill \\ {0\,\,\,\,\,\,\,\,\,\,\,\,\,\,\,\,\,\,\,\,\,\,\,\,\,\,\,\,\,\,\,\,\,\,\,\,\,\,\,\,\,\,\,\,\,\,\,\,\,\,\,\,\,\,\,\,{\text{if}}\,\,\,\,\,\,\,\,\,\,\,u_{Le} < 0\,,u_{Ri} > 0} \hfill \\ {\hat{d}_{{m + \tfrac{1}{2},n,p}} u_{Ri}^{2} \,\,\,\,\,\,\,\,\,\,\,\,\,\,\,\,\,\,\,\,\,\,\,\,\,\,\,\,\,\,\,\,\,\,\,\,\,{\text{if}}\,\,\,\,\,\,\,\,\,\,\,u_{Le} < u_{Ri} \,,\,u_{Ri} < 0} \hfill \\ \end{array} } \right.$$

In the correction step, the fluxes were calculated using a backward method as follows:

41 $$F_{m,n,p}^{c} = \left\{ {\begin{array}{*{20}l} {\hat{d}_{{m + \tfrac{1}{2},n,p}} .(u_{{m + \tfrac{1}{2},n,p}}^{p} )^{2} \,\,\,\,\,\,\,\,\,if\,\,\,\,\,\,\,\,\,u_{{m + \tfrac{1}{2},n,p}} + u_{{m - \tfrac{1}{2},n,p}} \ge 0} \hfill \\ {\hat{d}_{{m - \tfrac{1}{2},n,p}} .(u_{{m - \tfrac{1}{2},n,p}}^{p} )^{2} \,\,\,\,\,\,\,\,\,if\,\,\,\,\,\,\,\,\,u_{{m + \tfrac{1}{2},n,p}} + u_{{m - \tfrac{1}{2},n,p}} < 0} \hfill \\ \end{array} } \right.$$

To improve the precision of the flux calculations in the correction step, ${u}_{m+\frac{1}{2},n,p}^{p}$ used in Eq. (41) was computed using the following relation instead of Eq. (38):

42 $$\begin{gathered} \frac{{u_{{m + \tfrac{1}{2},n,p}}^{p} - u_{{m + \tfrac{1}{2},n,p}}^{n} }}{\Delta t} + \frac{1}{{d_{{m + \tfrac{1}{2},n,p}} }}\left( {\frac{{F_{m + 1,n,p}^{p} - F_{m,n,p}^{p} }}{\Delta x}} \right) - \frac{{u_{{m + \tfrac{1}{2},n,p}}^{n} }}{{d_{{m + \tfrac{1}{2},n,p}} }}\left( {\frac{{\phi_{m + 1,n,p}^{x} - \phi_{m,n,p}^{x} }}{\Delta x}} \right) + g\frac{\partial \eta }{{\partial x}} \hfill \\ \,\,\,\,\, + \frac{1}{{d_{{m + \tfrac{1}{2},n,p}} }}\left( {\frac{{\partial q^{n} }}{\partial x}} \right) = 0 \hfill \\ \end{gathered}$$

In this study, to suppress numerical oscillations and capture shocks around discontinuities, the SEA (Simple Efficient Algorithm) algorithm, which was introduced by Zia and Banihashemi^37^ and is considered a TVD (Total Variation Dimensioning) method, was used. This is achieved by using a flux limiter method and constraining part of the numerical flux in the discretized equation, numerical oscillations were prevented. In this method, the TVD fluxes were computed using the following relation:

43 $$\begin{gathered} F_{m,n,p} = \tfrac{1}{2}(F_{m,n,p}^{p} + F_{m,n,p}^{c} ) \hfill \\ F_{m,n,p} = F_{m,n,p}^{p} + \tfrac{1}{2}(F_{m,n,p}^{c} - F_{m,n,p}^{p} ) \hfill \\ F_{m,n,p}^{TVD} = F_{m,n,p}^{p} + \tfrac{1}{2}\psi (r)(F_{m,n,p}^{c} - F_{m,n,p}^{p} ) \hfill \\ \frac{{u_{{m + \frac{1}{2},n,p}}^{{\text{int}}} - u_{{m + \frac{1}{2},n,p}}^{n} }}{\Delta t} + \frac{1}{{d{}_{{m + \frac{1}{2},n,p}}}}\frac{{F_{m + 1,n,p}^{TVD} - F_{m,n,p}^{TVD} }}{\Delta x} = 0 \hfill \\ \end{gathered}$$

In the current research, the MUSCL (Monotonic Upstream-centered Schemes for Conservation Laws) limiter function^38^ was used:

44 $$\Psi (r) = \max \left[ {0,\min \left( {2,\tfrac{1}{2}r + \tfrac{1}{2},2r} \right)} \right]$$

where:

45 $$r = \left\{ {\begin{array}{*{20}l} {\frac{{F_{m - 1,n,p}^{c} - F_{m - 1,n,p}^{p} }}{{F_{m,n,p}^{c} - F_{m,n,p}^{p} }}\,\,\,\,\,\,\,\,\,\,\,\,\,{\text{if}}\,\,\,\,\,\,\,\,\,\,\,\,\,u_{{m + \tfrac{1}{2},n,p}} + u_{{m - \tfrac{1}{2},n,p}} \ge 0} \hfill \\ {\frac{{F_{m + 1,n,p}^{c} - F_{m + 1,n,p}^{p} }}{{F_{m,n,p}^{c} - F_{m,n,p}^{p} }}\,\,\,\,\,\,\,\,\,\,\,\,\,{\text{if}}\,\,\,\,\,\,\,\,\,\,\,\,u_{{m + \tfrac{1}{2},n,p}} + u_{{m - \tfrac{1}{2},n,p}} < 0} \hfill \\ \end{array} } \right.$$

The above procedure was used to calculate the flux resulting from the advection of velocity *u* in the *x*-direction (*F*^*ux*^). A similar procedure was used to calculate *F*^*uy*^, *F*^*vx*^, and *F*^*vy*^.

## Results and discussion

### Modeling of linear short-standing waves in a 3D closed basin

The first test employed to illustrate the effect of dynamic pressure distribution on the model results involved simulating linear short-standing wave oscillations within a 3D closed basin. For this purpose, a cubic basin with dimensions of 10 m in length, width, and depth was considered. The initial condition for the water surface elevation to form a standing wave can be expressed using the following relation:

46 $$\begin{gathered} \eta (x,y,t) = \frac{H}{2}\cos \left( {k_{x} .x} \right)\cos \left( {k_{y} .x} \right)\cos \left( {\omega .t} \right)\,\,\,\,\,\,\,\,\, \hfill \\ k_{x} = \frac{2\pi }{{L_{x} }},\,\,\,\,\,\,\,\,k_{y} = \frac{2\pi }{{L_{y} }}\,\,\,\,\,\,\,\,\,and\,\,\,\,\,\,\,\,\,\omega = \frac{2\pi }{T} \hfill \\ \end{gathered}$$

**Fig. 4:**
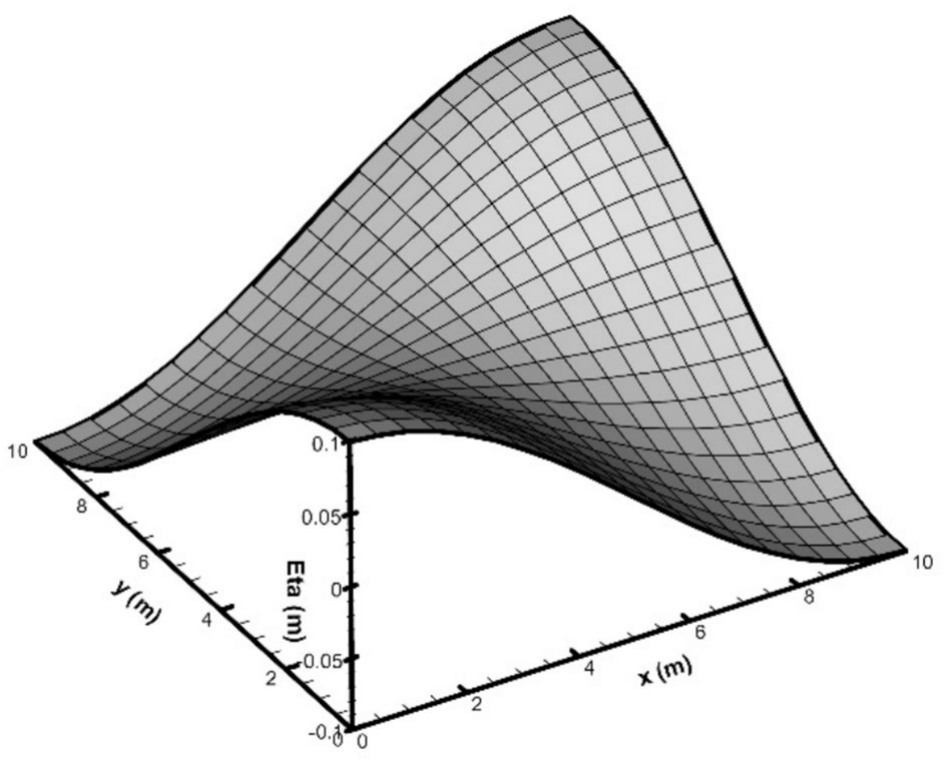
Initial elevation profile of the 3D standing wave in the closed basin.

In this test, the wavelength in each direction was considered to be twice the length of the basin, i.e., 20 m, with a wave height of 0.2 m. The wave period, calculated from the dispersion relation, was 3.01 s. The initial water surface profile at *t* = 0 is also shown in (Fig. 4). The dimensions of the computational grid in the horizontal directions, *Δx* and *Δy*, were set to 0.5 m, and five vertically uniform layers were used. The time step was configured to 0.05 s.

**Fig. 5:**
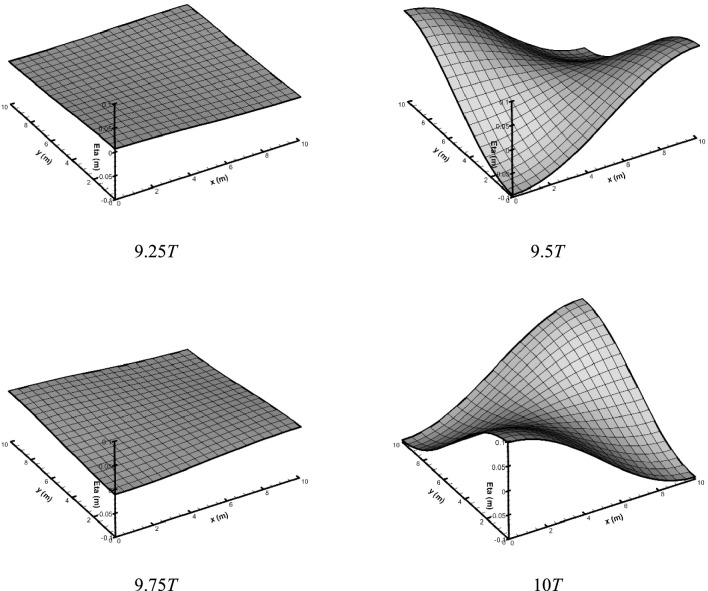
Water surface elevations obtained from the 3D numerical model at different times in the closed basin.

Figure 5 shows the water surface configurations from the 3D model at times 9.25, 9.5, 9.75, and 10* T*.

**Fig. 6:**
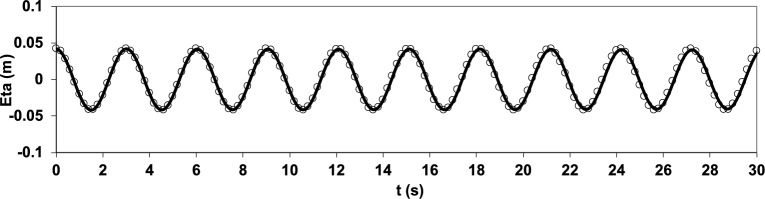
Comparison of water surface elevation from the numerical solution (solid line) and the analytical solution^39^ (circle) at the location *x* = 7.25 m and *y* = 7.25 m.

The results of this simulation are presented as a comparison of the water surface elevation from the numerical model with that from the analytical solution at the location *x* = 7.25 m and *y* = 7.25 m, as shown in (Fig. 6). The details of the analytical solution can be found in Dean and Dalrymple^39^. In this test, given the ratio $h=\surd 2\pi$, deep water conditions were established, and the vertical acceleration component played a significant role. The accuracy of vertical acceleration predictions is influenced by the number of vertical layers and how the pressure is handled in the uppermost cell. The results highlight the model’s ability to simulate linear short-standing wave oscillations in deep water using only 20 cells in the longitudinal direction and five vertical layers.

**Fig. 7:**
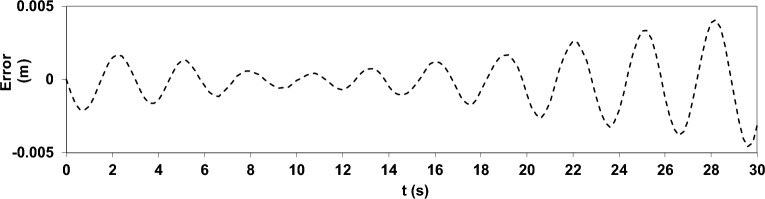
Temporal residuals (numerical – analytical) of the standing wave test (corresponding to Fig. 6).

To further highlight the deviations between the numerical and analytical solutions, the residual was calculated as the difference between the two solutions at each time step and its temporal variation was plotted. The corresponding residual plot is presented in (Fig. 7).

As illustrated in Fig. 7, the residual remains very small throughout the simulation, with values confined within ± 0.005 m. The oscillatory pattern of the error indicates that the deviations are mainly due to the accumulation of numerical discretization effects over time, rather than a systematic bias. Moreover, the residual oscillates around zero, confirming that the numerical model neither consistently underestimates nor overestimates the analytical solution. Overall, the residual plot confirms the high accuracy and reliability of the present model in reproducing the analytical results.

### Modeling of sine wave propagation over an elliptical submerged mound

**Fig. 8:**
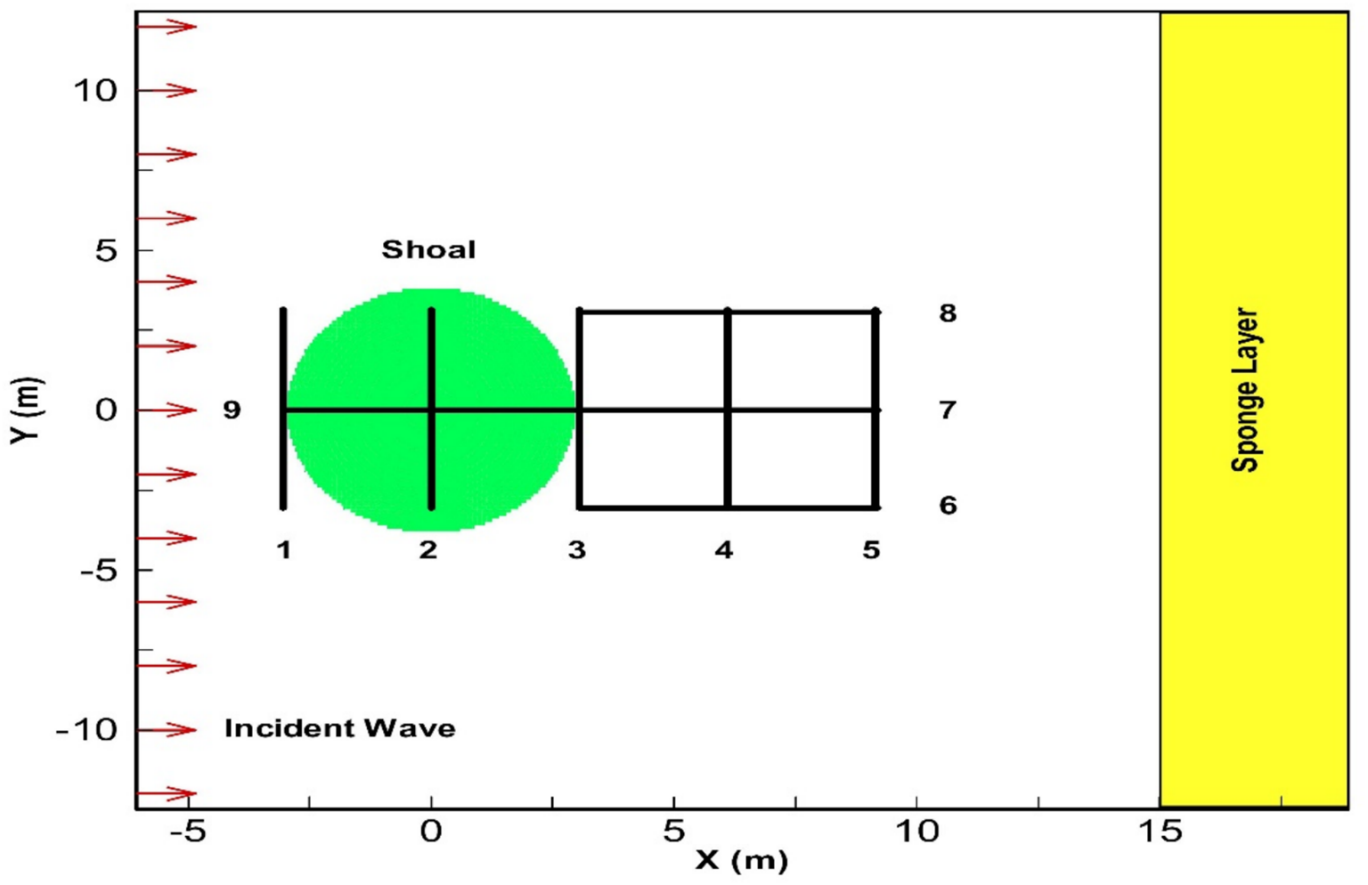
Measurement sections and specifications of the laboratory test by Vincent and Briggs^40^ for wave propagation over an elliptical submerged mound.

This test aims to evaluate how effectively the 3D simulation captures phenomena like wave shoaling, refraction, and diffraction, and to explore how nonlinear effects interact with dynamic pressure. To achieve this, the numerical model results were validated against experimental data from Vincent and Briggs^40^. In this experiment, a propagating wave travels over a variable topography created by an elliptical submerged mound. Figure 8 shows the geometry of this test, including the coordinate system, topography, and locations for output data collection. The computational domain extends from *x* =  − 6.1 m to *x* = 18.9 m and from *y* =  − 12.5 m to *y* = 12.5 m.

According to the provided coordinates, the elliptical mound was positioned using the following relation:

47 $$\left( {\frac{x}{3.05}} \right)^{2} + \left( {\frac{y}{3.96}} \right)^{2} = 1$$

The still water depth outside the elliptical boundary is constant at 0.457 m, and inside this boundary, it was calculated using the following relation:

48 $$h = 0.9144 - 0.762\sqrt {1 - \left( {\frac{x}{3.81}} \right)^{2} - \left( {\frac{y}{4.95}} \right)^{2} }$$

**Fig. 9:**
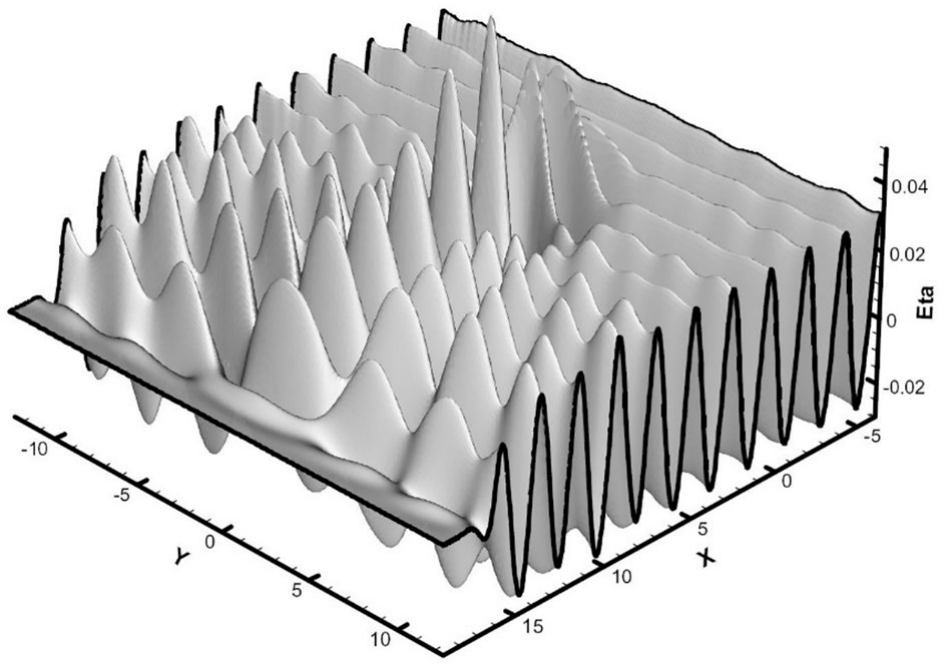
Final and 3D state of the wave field after the simulation period.

In this test, a wave with a height of *H*_*0*_ = 2.54 cm and a period of 1.3 s enters from the western boundary (left side), with a *kh* of 1.27. Therefore, in the numerical model, the depth-wise discharge distribution was considered based on linear theory for the incoming wave. At the right boundary, both a radiation BC and a sponge layer were used to reduce wave reflection, while reflective BCs were applied at the lateral boundaries. The computational domain was discretized with grid spacings of ∆x = 0.05 m and ∆y = 0.1 m, using two vertical layers where the lower layer accounted for 70% and the upper layer for 30% of the total depth. The time step was 0.02 s, and the overall duration of the simulation was selected to satisfy stability requirements. The final wave field configuration is shown in (Fig. 9).

**Fig. 10:**
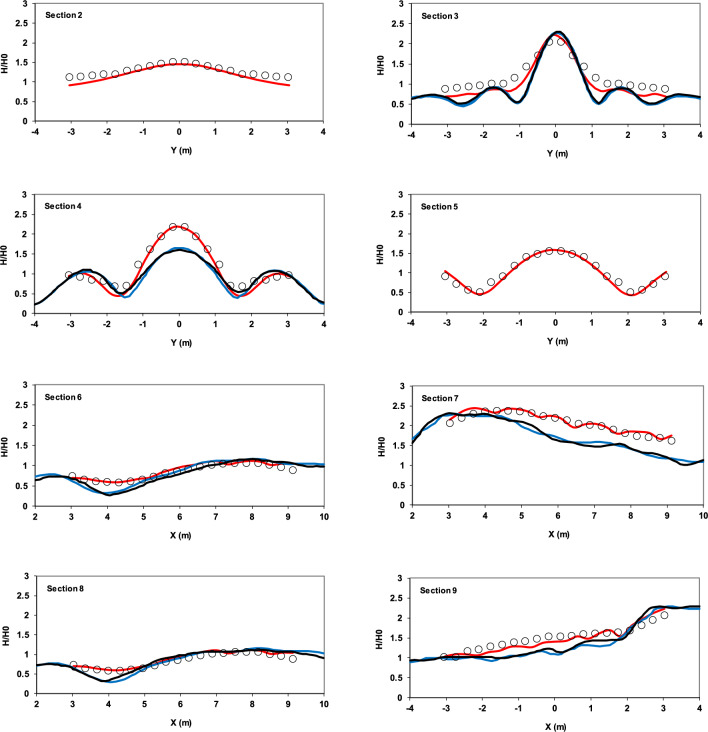
Comparison of dimensionless wave height for sinusoidal wave propagation over an elliptical submerged mound at specified sections between experimental data from Vincent and Briggs^40^ (circles), results of the current model (red lines), results of the Fang et al.^41^ model (blue lines), and results of the Knag and Guo^42^ model (black lines).

The dimensionless wave height at eight designated sections was compared with experimental data (Fig. 10). The wave height was measured over the last five periods.

To further evaluate the performance of the present numerical model, its results were compared with those from two well-established models that have demonstrated reliable performance against experimental data from Vincent and Briggs^40^. The model developed by Fang et al.^41^ is a finite volume-based, shock-capturing scheme that solves the governing equations using a fractional-step method, in which the total pressure is decomposed into hydrostatic and non-hydrostatic components. While the hydrostatic part is integrated explicitly, the non-hydrostatic part is selectively deactivated near wave fronts to represent breaking waves as shocks. On the other hand, the model proposed by Kang and Guo^42^ employs an alternating direction implicit (ADI) scheme in a depth-integrated non-hydrostatic framework. Each time step is split into two half-steps, in which dynamic pressure and velocity components are discretized implicitly in one direction and explicitly in the other. A tridiagonal solver (Thomas algorithm) is used to efficiently solve the resulting linear systems.

The analysis of the results demonstrates the model’s ability to simulate the phenomena of interest. In particular, the accuracy of the wave shoaling simulation is evident in Sect. 9 and, additionally, in Sect. 7. The wave refraction phenomenon in front of the mound in Sect. 3 is observed, with wave concentration at the center of this section. Furthermore, in Sect. 4, due to diffraction, the wave energy disperses towards the edges. Table 2 clearly demonstrates that the present model outperforms the other two numerical models in terms of accuracy, as evidenced by its lower RMSE values at all measured sections.

**Table 2:** RMSE comparison of three numerical models against Vincent and Briggs^40^ experimental data.

Section ^40^	Kang and Guo^41^ (RMSE)	Fang et al.^42^ (RMSE)	Present model (RMSE)
Section 2	–	–	0.100
Section 3	0.297	0.306	0.165
Section 4	0.284	0.305	0.130
Section 5	–	–	0.090
Section 6	0.132	0.131	0.070
Section 7	0.366	0.365	0.092
Section 8	0.124	0.138	0.071
Section 9	0.231	0.260	0.109

In addition to the RMSE values reported in (Table 2), the mean absolute error (MAE) and mean bias error (MBE) were calculated to provide further insight into the model performance. These results are summarized in (Table 3).

**Table 3:** MAE and MBE of the numerical model compared with experimental data^40^.

Section ^40^	MAE	MBE
Section 2	0.078	−0.078
Section 3	0.158	−0.114
Section 4	0.108	−0.058
Section 5	0.071	−0.010
Section 6	0.056	0.052
Section 7	0.076	0.061
Section 8	0.060	0.056
Section 9	0.098	−0.018

The computed MAE and MBE values indicate that the model accuracy in reproducing the experimental results depends strongly on the section location relative to the elliptical mound. The largest MAE occurs at Sect. 3 (0.158), where strong wave shoaling, refraction, and diffraction lead to energy focusing and enhanced local variability. The negative MBE (− 0.114) at this section shows a tendency of the model to underestimate the wave height compared with the measurements. Similarly, in Sect. 4, where wave diffraction redistributes energy toward the flanks, the MAE remains relatively high (0.108). In contrast, the lowest errors are observed at Sects. 6 (MAE = 0.056) and 8 (MAE = 0.060), located perpendicular to the wave propagation direction, where the wave field is more uniform. The positive MBE values at these Sects. (0.052 and 0.056) suggest a slight overestimation of wave heights by the model. Overall, the results confirm that errors are more pronounced in regions strongly affected by refraction and diffraction, while they decrease in more regular wave zones. Nevertheless, the generally low MAE values (mostly below 0.16) and small MBE magnitudes across the sections demonstrate the robustness and reliability of the present 3D model in capturing the complex wave transformations over the submerged elliptical mound.

### Modeling wave propagation over an elliptical mound on a sloping bed

This test is similar to the previous one but with the elliptical mound situated on a sloping bed rather than a flatbed. The increased incoming wave height and shorter period compared to the previous test leads to a more pronounced interaction between nonlinear behavior and dynamic pressure. Therefore, this test provides a more comprehensive assessment of the simulation’s ability to model phenomena such as wave shoaling, diffraction, and refraction.

**Fig. 11:**
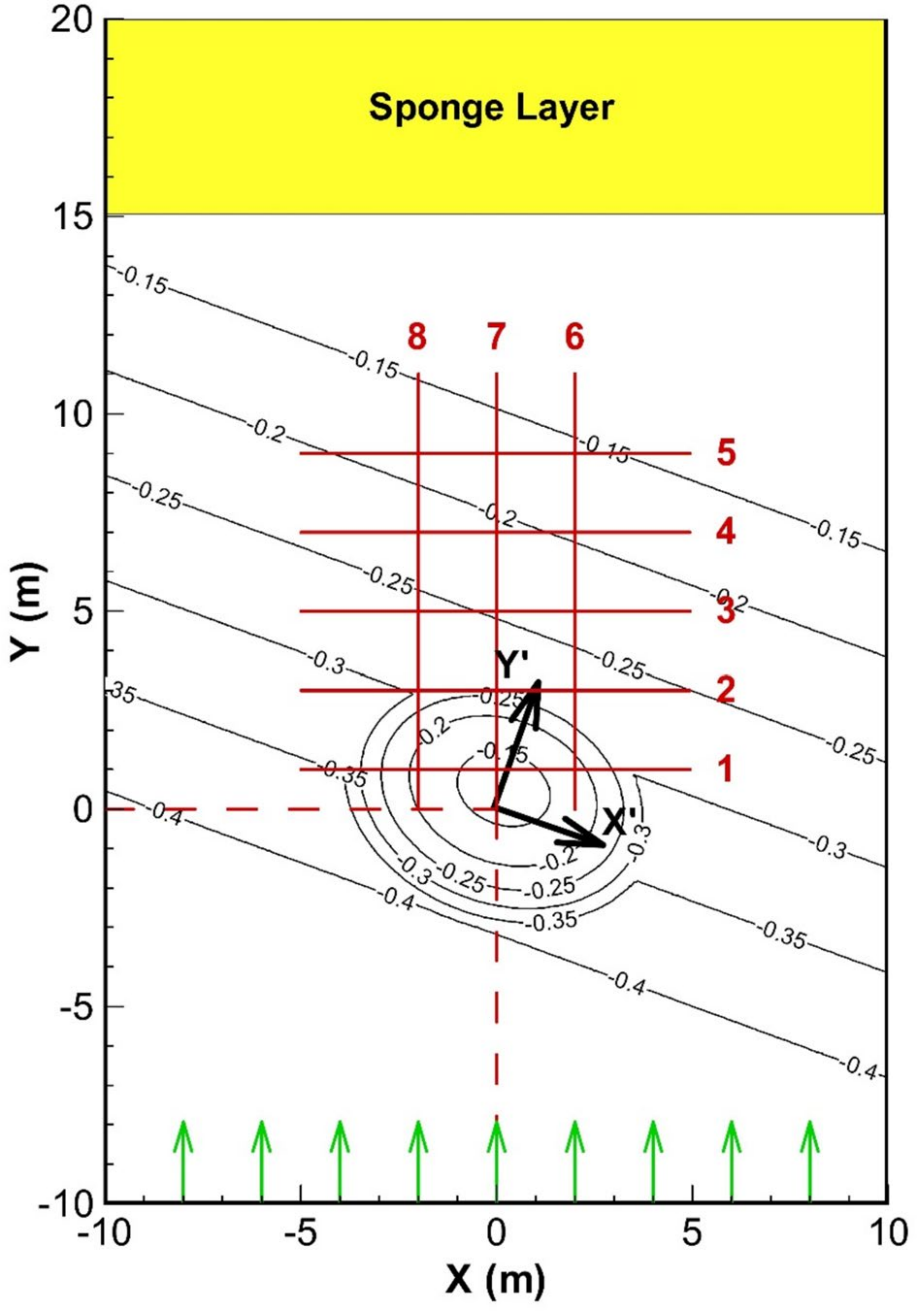
Measurement sections and specifications of the laboratory test by Berkhoff et al.^43^ for wave propagation over an elliptical mound on a sloping bed.

The numerical model results were compared with experimental data from Berkhoff et al.^43^. According to Fig. 11, in this experiment, a regular wave propagates over an elliptical mound situated on a bed with a slope of 1:50. A secondary coordinate frame (x′, y′) is oriented at -20 degrees relative to the primary coordinate system. In this local reference frame, the *x*′ axis aligns with the shore, while the *y*′ axis is oriented perpendicular to the shore. In this case, the still water depth, excluding the elliptical mound, is calculated using the following relation:

49 $$\begin{gathered} h = 0.45\,\,\,\,\,\,\,\,\,\,\,\,\,\,\,\,\,\,\,\,\,\,\,\,\,\,\,\,\,\,\,\,\,\,\,\,\,\,\,\,\,\,\,\,\,\,\,\,\,\,\,\,\,\,\,\,\,\,\,\,\,\,\,\,\,\,\,\,\,\,\,\,\,\,\,\,\,\,\,\,\,\,\,\,\,\,{\text{for}}\,\,\,\,\,\,\,\,y^{\prime} < - 5.484 \hfill \\ h = \max \left[ {0.1,\,\,0.45 - 0.02(5.484 + y^{\prime})} \right]\,\,\,\,\,\,\,\,\,\,\,\,\,\,\,\,\,\,\,\,\,\,\,\,{\text{for}}\,\,\,\,\,\,\,\,y^{\prime} \ge - 5.484 \hfill \\ \end{gathered}$$

The boundary of the elliptical mound is specified using the following relation:

50 $$\left( {\frac{{x^{\prime}}}{4}} \right)^{2} + \left( {\frac{{y^{\prime}}}{3}} \right)^{2} = 1$$

and the mound thickness is obtained using the following relation:

51 $$d_{s} = - 0.3 + 0.5\sqrt {1 - \left( {\frac{{x^{\prime}}}{5}} \right)^{2} - \left( {\frac{{y^{\prime}}}{3.75}} \right)^{2} }$$

In this test, a regular wave with a height of 4.64 cm and a period of one second enters the computational domain from the southern boundary (*y* = -10 m).

**Fig. 12:**
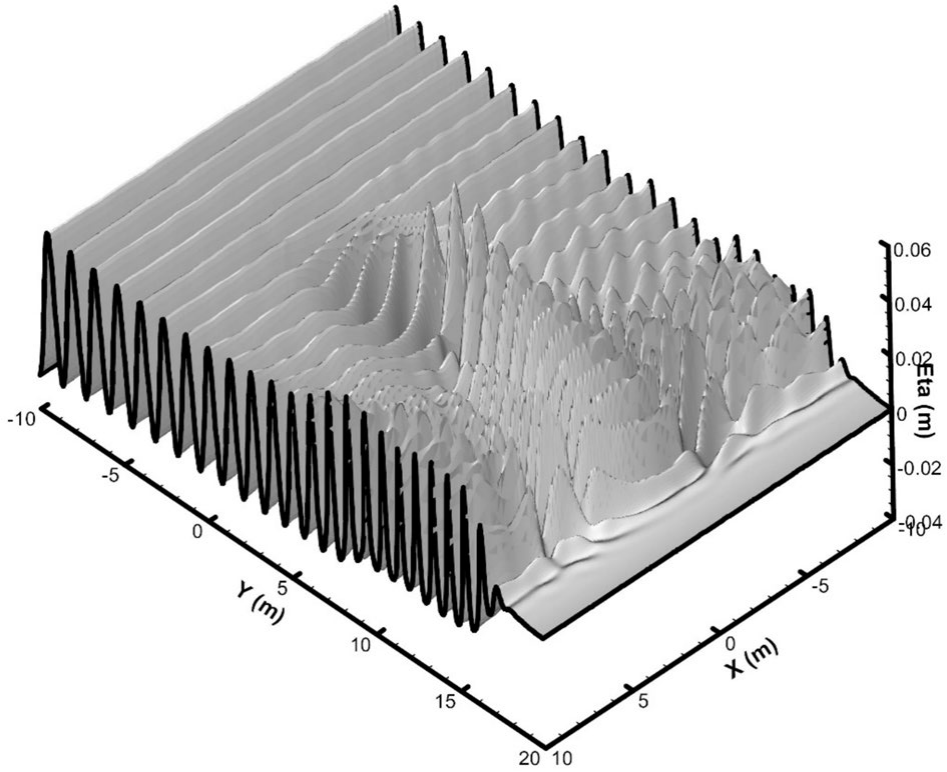
3D water surface elevation of the wave field after the simulation period.

In the simulation, the wave loading was represented as a depth-wise discharge distribution based on linear wave theory. At the boundary (*y* = 20 m), a 5-m-long sponge layer and radiation BC were used to absorb the waves. For the side boundaries (*x* = -10 m and *x* = 10 m), reflective wall conditions were used, similar to those in the laboratory model. The computational domain was divided using grid intervals of 0.1 m in the *x* direction and 0.05 m in the *y* direction. The domain was segmented into two vertical layers, with the bottom layer covering 30% and the top layer covering 70% of the total depth. A time step of 0.01 s was utilized. The simulation continued until equilibrium was achieved. The final 3D profile of the water surface across the entire domain is presented in (Fig. 12).

**Fig. 13:**
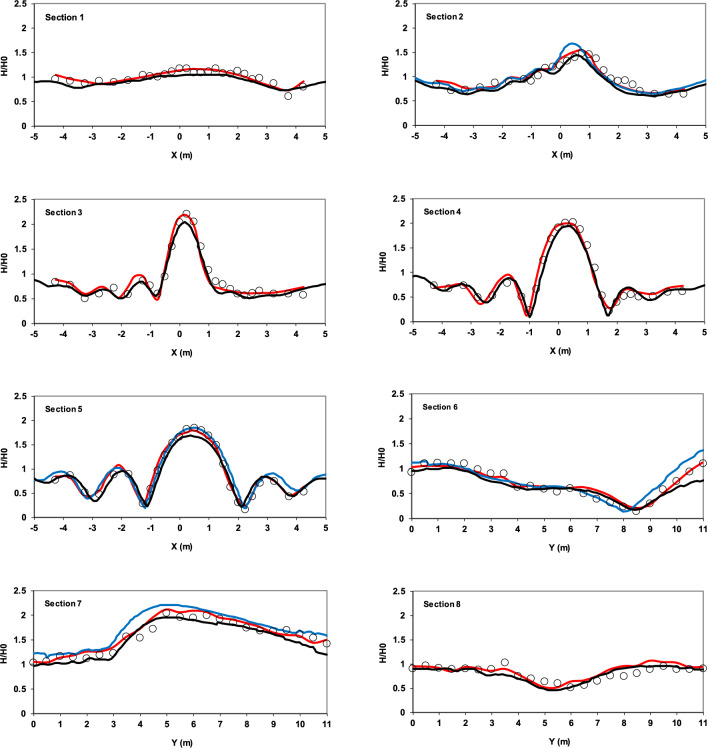
Comparison of dimensionless wave heights for sinusoidal wave propagation over an elliptical mound on a sloping bed at specified sections, between experimental data from Berkhoff et al.^43^ (circles), results of the current model (red lines), results of the Stelling and Zijlema^44^ model (blue lines), and results of the Ma et al.^45^ model (black lines).

The dimensionless wave height at eight specified sections in Fig. 11 were compared with experimental data. The wave height was determined by averaging the values over the last five periods after the solutions had stabilized. In Fig. 13, the comparison between the numerical model results and experimental data for dimensionless wave height with the incoming wave is presented.

Two well-established models that have used these tests for validation are the Stelling and Zijlema^44^ model and the Ma et al.^45^ model. Stelling and Zijlema^44^ employ the Keller-box implicit scheme, which approximates non-hydrostatic vertical pressure gradients at different vertical grid points simultaneously. In the Ma et al.^45^ model, NHWAVE, a shock-capturing non-hydrostatic model for simulating nonlinear free-surface wave processes, was introduced. The governing equations were solved in a σ-coordinate system. To highlight the significance of wave refraction and diffraction, Stelling and Zijlema^44^ compared their numerical results with measured data from Berkhoff et al.^43^ at four sections, while Ma et al.^45^ carried out the comparison at all sections. To validate the accuracy of the present model, the results obtained from the Stelling and Zijlema^44^ and Ma et al.^45^ models are also presented in (Fig. 13).

Based on the phenomenon of refraction, wave concentration in front of the mound at Sect. 3 reaches a maximum, with a wave height 2.2 times that of the incoming wave. As the wave moves away from the mound, diffraction becomes apparent, particularly in Sects. 4 and 5.

The RMSE analysis shows that the present numerical model achieves higher accuracy in estimating dimensionless wave height compared to the other two numerical models (Table 4). For the present model, the RMSE values at Sects. 1 to 8 are 0.060, 0.096, 0.114, 0.107, 0.084, 0.077, 0.101, and 0.105, respectively. In comparison, the corresponding values for the numerical model by Ma et al.^45^ are 0.086, 0.128, 0.110, 0.099, 0.104, 0.129, 0.120, and 0.100. For the model by Stelling and Zijlema^44^, the RMSE values at Sects. 2, 5, 6, and 7 are 0.134, 0.115, 0.128, and 0.205, respectively. These results demonstrate that the present model provides more accurate predictions than the model by Stelling and Zijlema^44^ across all evaluated sections and outperforms Ma et al.^45^ in most sections, with only minor differences in accuracy for the remaining sections.

**Table 4:** RMSE comparison of three numerical models against Berkhof et al.^43^ experimental data.

Section ^43^	Stelling and Zijlema^44^ (RMSE)	Ma et al.^45^ (RMSE)	Present model (RMSE)
Section 1	−	0.086	0.060
Section 2	0.134	0.128	0.096
Section 3	−	0.110	0.114
Section 4	−	0.099	0.107
Section 5	0.115	0.104	0.084
Section 6	0.128	0.129	0.077
Section 7	0.205	0.120	0.101
Section 8	−	0.100	0.105

To further quantify the model accuracy, the mean absolute error (MAE) and mean bias error (MBE) were also computed for the measured sections, and the results are presented in (Table 5).

**Table 5:** MAE and MBE of the numerical model compared with experimental data^43^.

Section ^43^	MAE	MBE
Section 1	0.049	0.019
Section 2	0.080	0.024
Section 3	0.096	0.007
Section 4	0.090	0.026
Section 5	0.071	0.025
Section 6	0.061	0.013
Section 7	0.081	0.059
Section 8	0.088	0.050

The MAE and MBE values highlight the dependence of model accuracy on the section location relative to the elliptical mound on the sloping bed. The lowest error is found at Sect. 1 (MAE = 0.049), where the wave field remains more uniform and less affected by refraction or diffraction, resulting in close agreement between numerical and experimental results. The largest errors occur at Sects. 3 (MAE = 0.096) and 4 (MAE = 0.090), which coincide with energy focusing due to shoaling and refraction in front of the mound and energy redistribution caused by diffraction toward the flanks. These regions pose greater challenges for numerical reproduction of wave transformations. Downstream of the mound, the errors decrease (e.g., MAE = 0.071 at Sect. 5), reflecting improved model–data agreement as the wave field becomes more regular. A slight increase in errors at Sects. 7 (0.081) and 8 (0.088) may be attributed to residual divergence effects in the far field. The MBE values are consistently small and positive (up to 0.059 at Sect. 7), indicating a mild tendency of the model to overestimate wave heights. Overall, the generally low MAE (< 0.1) and small MBE values confirm the robustness of the present 3D model in capturing wave shoaling, refraction, and diffraction processes over the submerged mound on a sloping bed.

### Modeling of long wave resonance in a parabolic basin

To evaluate the model performance in handling wet/dry conditions in a 2D plan, an existing analytical solution for a nonlinear shallow water problem was used. In this context, a long wave oscillation test in a parabolic basin was chosen, providing a rigorous and challenging test for numerical models. In this test, assuming that the coordinate system’s origin is at the center of the basin, the bed of the parabolic basin is defined by the following relation:

52 $$z_{b} = - h_{0} \left( {1 - \frac{{r^{2} }}{{R^{2} }}} \right)\,\,\,,\,\,\,r = \sqrt {x^{2} + y^{2} }$$

Thacker^46^ provides the analytical formula for the water surface height (*η*) and the radial horizontal velocity (*u*_*r*_), derived from the nonlinear shallow water equations, as follows:

53 $$\eta_{r,t} = h_{0} \left[ {\frac{{\sqrt {1 - A^{2} } }}{1 - A.\cos \omega \,t} - 1 - \frac{{r^{2} }}{{R^{2} }}\left( {\frac{{1 - A^{2} }}{{(1 - A.\cos \omega \,t)^{2} }} - 1} \right)} \right]$$

54 $$u_{r,t} = \frac{\omega .r.A.\sin \omega \,t}{{2(1 - A.\cos \omega \,t)}}\,\,\,,\,\,\,A = \frac{{(R^{4} - r_{0}^{4} )}}{{(R^{4} + r_{0}^{4} )}}\,\,\,,\,\,\,\omega = \frac{1}{R}\sqrt {8gh_{0} }$$

The numerical values for the parameters used in this test were set as *h*_*0*_ = 1 m, *r*_*0*_ = 2000 m, and *R* = 2500 m. Therefore, the 2D computational domain was selected from *x* =  − 3500 m to *x* = 3500 m and *y* =  − 3500 m to *y* = 3500 m, using a grid spacing of *Δx* = *Δy* = 20 m. The vertical direction utilized two layers of equal thickness, and a time step of 2 s was selected. Based on the analytical solution, the flow was considered frictionless. A minimum depth value of 10^−5^ m was used to establish wet/dry conditions. The initial water surface elevation in this test was determined by setting *t* = 0 in Eq. (53), with the velocity values being zero under these conditions. Since this test involves long waves, solving the pressure gradient term is not necessary, and thus it is not included in the numerical model. The exclusion of the pressure gradient term is equivalent to assuming a hydrostatic pressure distribution. Additionally, the temporal weighting factor *θ* in Eq. (20) was set to 0.5 to ensure that the calculations achieved second-order temporal accuracy.

**Fig. 14:**
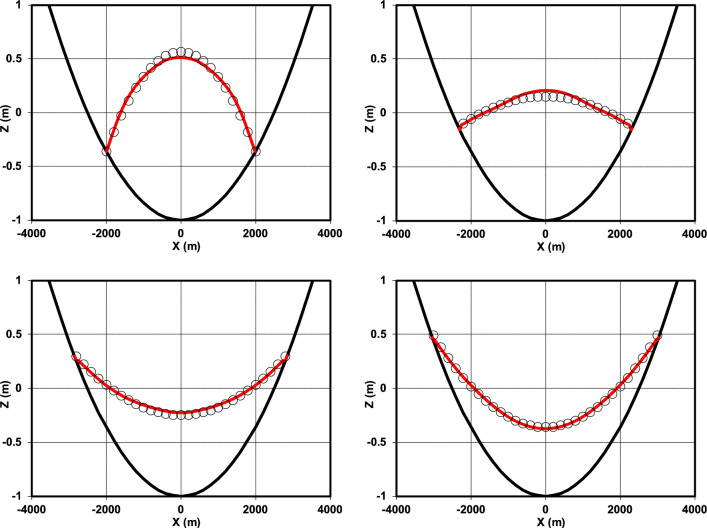
Comparison of the water surface elevation at the cross-section *y* = 0 and at times *t* = *T* (upper left), *t* = 7* T*/6 (upper right), *t* = 4* T*/3 (lower left), and *t* = 3* T*/2 (lower right), showing the analytical solution^46^ (circles) versus the numerical solution (red line), with the bed level indicated by the black line.

**Fig. 15:**
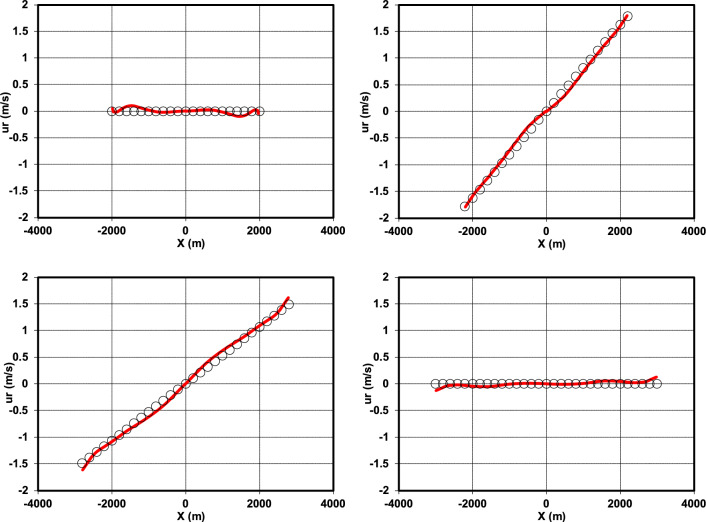
Comparison of the radial velocity at the cross-section *y* = 0 and at times *t* = *T* (upper left), *t* = 7* T*/6 (upper right), *t* = 4* T*/3 (lower left), and *t* = 3* T*/2 (lower right), showing the analytical solution^46^ (circles) versus the numerical solution (red line).

The comparison between the numerical solution and the analytical solution of the water surface elevation at the cross-section *y* = 0 after one period is shown in (Fig. 14). Additionally, a comparison of the radial velocity from the numerical and analytical approaches under the specified conditions was conducted, with the findings illustrated in (Fig. 15).

In addition to the visual comparison shown in (Fig. 14), the RMSE was computed at different phases of the oscillation to better quantify the model accuracy. These results are presented in (Table 6).

**Table 6:** RMSE of the numerical model for water surface elevation at different time instants (Fig. 14).

*t*	*T*	7* T*/6	4* T*/3	3* T*/2
RMSE	0.032	0.029	0.021	0.014

The RMSE values demonstrate excellent agreement between the numerical and analytical solutions at all time instants (all below 0.04). Slightly higher errors are observed at *t* = *T* (0.032) and *t* = 7* T*/6 (0.029), which can be attributed to the stronger variations in free-surface elevation during these phases. As the oscillation progresses, the RMSE decreases, reaching its minimum value at *t* = 3* T*/2 (0.014). This decreasing trend indicates that the model achieves higher accuracy in reproducing more stable and uniform flow patterns.

To further evaluate the model performance, the RMSE values of the radial velocity shown in (Fig. 15) were calculated at different time instants. The results are presented in (Table 7).

**Table 7:** RMSE of the numerical model for radial velocity at different time instants (Fig. 15).

*t*	*T*	7* T*/6	4* T*/3	3* T*/2
RMSE	0.049	0.065	0.063	0.047

The RMSE values confirm that the model reproduces the velocity field with good accuracy, although the errors are slightly larger than those for water surface elevation . The highest errors occur at *t* = 7* T*/6 (0.065) and *t* = 4* T*/3 (0.063), corresponding to phases of rapid flow variation where pressure gradients and local accelerations are strongest. In contrast, lower errors are found at *t* = *T* (0.049) and *t* = 3* T*/2 (0.047), which represent more stable phases of the oscillation. A comparison with Fig. 14 highlights a different error evolution between the two variables. While the RMSE for water surface elevation (Fig. 14) decreases monotonically over time, the RMSE for radial velocity (Fig. 15) first increases from *t* = *T* to *t* = 7* T*/6 and then decreases toward *t* = 3* T*/2. This behavior reflects the higher sensitivity of the velocity field to transient flow variations and pressure gradients, whereas the free-surface elevation exhibits a more uniform and stable trend during the oscillation.

Overall, the consistently low RMSE values (all below 0.07) in (Figs. 14, 15) demonstrate the robustness of the present model, which in these cases employs only two vertical layers, in simultaneously reproducing both the free-surface oscillations and the velocity field in the parabolic basin, while maintaining numerical stability throughout the simulation.

However, it should be noted that accuracy deteriorates after several periods. This reduction in accuracy results from the use of a Cartesian grid for circular tests—a limitation documented in previous studies for Boussinesq models^47–49^ and for non-hydrostatic models^33,50^. However, the change in water volume after 10 periods is on the order of 10^−7^, with $\frac{{V}_{10T}-{V}_{0}}{{V}_{0}}\approx {10}^{-7}$.

## Conclusion

In this research, the 3D RANS equations are discretized with the finite volume approach on a standard staggered grid system. The governing equations are tackled using a pressure-correction method with a time-splitting technique, carried out in two main phases. First, the intermediate velocities are computed by solving the advection and diffusion terms, gradient of the water surface and the explicit dynamic pressure terms in the momentum equations, applying a time-splitting method and suitable techniques for each component. Recognizing the importance of horizontal advection terms in wave propagation simulations, a Godunov-type shock-capturing scheme was utilized to address these aspects. Furthermore, a novel approach is introduced for estimating horizontal velocities at vertical velocity locations, which enhances the sparsity of the coefficient matrix in the pressure equation and reduces overall computational complexity in three-dimensional applications. Next, these intermediate velocities, together with the gradient of the pressure adjustment term from the momentum equations, are incorporated into the conservation of mass equation. This process results in the formulation of a Poisson equation for pressure adjustment by eliminating the velocity components. The strong agreement between the simulation results, experimental data, and analytical solutions demonstrates the capability of the model to accurately reproduce nonlinear wave phenomena such as shoaling, refraction, and diffraction. Owing to its accuracy, flexibility, and reduced computational demand, the model offers a practical and valuable tool for analyzing complex free-surface flows and supporting engineering decisions in coastal and environmental water systems. It should be acknowledged, however, that the present study has focused on non-breaking wave propagation, and the explicit treatment of wave breaking processes has not been addressed. Future research will aim to extend the developed framework toward fully three-dimensional modeling of breaking waves, thereby broadening its applicability to more complex coastal hydrodynamic conditions.

## Data Availability

The datasets used and/or analysed during the current study available from the corresponding author on reasonable request.

## List of symbols

*P*: Total pressure (Pa)
*g*: Gravitational acceleration (m s^−^^2^)
$x$: Coordinate along the $x$-axis in a Cartesian coordinate system (m)
$y$: Coordinate along the $y$-axis in a Cartesian coordinate system (m)
$z$: Coordinate along the $z$-axis in a Cartesian coordinate system (m)
*q*: Dynamic pressure divided by density (Pa m^3^ kg^−^^1^)
*u*: Velocity component along the $x$-axis (m s^−^^1^)
*v*: Velocity component along the $y$-axis (m s^−^^1^)
*w*: Velocity component along the $z$-axis (m s^−^^1^)
*t*: Time (s)
*h*: Still water depth (m)
*P*_*a*_: Atmospheric pressure (Pa)
$\Delta x$: Cell size along the $x$-axis (m)
$\Delta y$: Cell size along the $y$-axis (m)
$\Delta q$: Pressure correction term divided by density (Pa m^3^ kg^−^^1^)
*D*: Total water depth (m)
*d*: The thickness of each layer (m)
$f_{p}$: Kth layer thickness coefficient (−)
$\widehat{D}$: Water depth next to the cell boundary (m)
*U*: Depth-averaged u (m s^−^^1^)
*V*: Depth-averaged v (m s^−^^1^)
*F*: Advection flux (m^3^ s^−^^2^)
*u*^*int*^: Intermediate velocity component in the $x$-direction (m s^−^^1^)
*v*^*int*^: Intermediate velocity component in the $y$-direction (m s^−^^1^)
*w*^*int*^: Intermediate velocity component in the $z$-direction (m s^−^^1^)
*u*^*adv*^: Advected $x$-velocity (m s^1^)
*v*^*adv*^: Advected $y$-velocity (m s^1^)
*w*^*adv*^: Advected $z$-velocity (m s^1^)
*T*: Period (s)
$$\Delta t$$: Time step (s)
$\hat{d}$: Cell-edge layer thickness (m)
*b*_*1*_*, b*_*2*_*, and b*_*3*_: Weighting coefficients determined by the weighted layer thickness (−)
*H*: Wave height (m)
*k*: Wave number (m^−^^1^)
*L*: Wavelength (m)
*H*_*0*_: Incoming wave height (m)
${\acute{\rm \text{x}}}$: Horizontal coordinate along the ${\acute{\rm \text{x}}}$-axis in the local coordinate framework (m)
${\acute{\rm \text{y}}}$: Horizontal coordinate along the ${\acute{\rm \text{y}}}$-axis in the local coordinate framework (m)
*e*: Interpolation coefficient
*d*_*s*_: Mound thickness (m)
*z*_*b*_: Bed elevation coordinate of the parabolic basin (m)
*h*_*0*_: Depth of still water at the basin’s center (m)
*r*: Distance from the center of the basin (m)
*R*: The radius of the still water level (m)
*r0*: Radius from the center of the basin to a still water level contour (m)
*V*_*10T*_: Water volume after ten periods (m^3^)
*V*_*0*_: Initial water volume (m^3^)

## Greek symbols

$\rho$: Mass density (kg m^−^^3^)
$\eta$: Water surface elevation (m)
*V*_*hor*_: Horizontal eddy viscosity (m^2^ s^−^^1^)
*V*_*ver*_: Vertical eddy viscosity ( m^2^ s^−^^1^)
$\rho 0$: Water density (kg m^−^^3^)
$$\psi (r)$$: Limiter function (−)
$\phi$: Flux through each layer (m^2^ s^−^^1^)
$\xi$: Relative vertical velocity (m s^−^^1^)
$\theta$: The weighting factor for the time discretization approach (−)
$\omega$: Angular frequency of the wave (^−^^1^)

## Subscripts

$x$: Along the *x*-axis
$y$: Along the *y*-axis
*m*: Cell center index along the *x*-axis
*n*: Cell center index along the *y*-axis
*p*: Cell center index along the *z*-axis
*T*: Position for obtaining the vertical gradient of dynamic pressure in the upper half of the surface layer
*np*: Number of layers
*s*: Surface level
*Le*: The left side of the computational cell wall
*Ri*: The right side of the computational cell wall
*r,t*: At a distance r from the center of the mound and at time t
*: Intermediate velocity

## Superscripts

*n*: Time-step counter
*p*: Related to the prediction phase
*c*: Related to the correction phase
*TVD*: Related to the TVD approach
*x*: Along the *x*-axis
*y*: Along the *y*-axis
*ux(uy), vx(vy)*: Flux of u along the *x (y)* direction, Flux of v along the *x (y)* direction
